# Depletion sphere: Explaining the number of Ag islands on Au nanoparticles†
†Electronic supplementary information (ESI) available: Details of the synthesis; UV-Vis and SERS spectra; and large-view TEM images. See DOI: 10.1039/c6sc02276f



**DOI:** 10.1039/c6sc02276f

**Published:** 2016-08-17

**Authors:** Yuhua Feng, Yawen Wang, Xiaohui Song, Shuangxi Xing, Hongyu Chen

**Affiliations:** a Institute of Advanced Synthesis (IAS) , School of Chemistry and Molecular Engineering , Jiangsu National Synergetic Innovation Center for Advanced Materials , Nanjing Tech University , Nanjing 211816 , P. R. China . Email: iashychen@njtech.edu.cn; b Division of Chemistry and Biological Chemistry , School of Physical and Mathematical Sciences , Nanyang Technological University , Singapore 637371; c Faculty of Chemistry , Northeast Normal University , Changchun 130024 , P. R. China . Email: xingsx737@nenu.edu.cn

## Abstract

We report multi-site nucleation and growth of Ag islands on colloidal Au nanoparticles. By modifying a single factor, a range of products from Janus nanoparticles to satellite nanostructures are obtained. The identification of these key factors reveals the correlation between the concentration gradient and the choice of nucleation sites. In contrast to the inhibited homogeneous nucleation in the bulk solution, we argue that the non-steady-state concentration gradient plays a critical role in inhibiting nucleation within nanometer distance during the initial stage of growth—an essential but not yet recognized factor in colloidal synthesis. A depletion sphere model was developed, so that the multi-site nucleation is well integrated with the classic theory of nucleation and growth. Alternative explanations are carefully examined and ruled out. We believe that the synthetic know-how and the mechanistic insights can be broadly applied and are of importance to the advance of nanosynthesis.

## Abstract 2

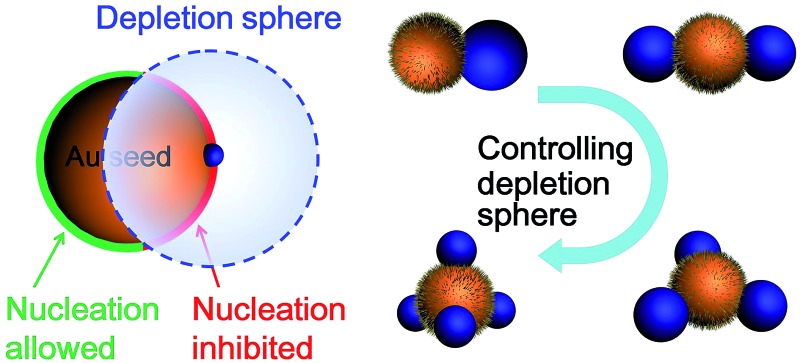
We propose the depletion sphere model to explain the control of valency when forming Au–Ag satellite nanostructures.

## Introduction

Nanoparticles are the basic building blocks of nanotechnology. Despite tremendous synthetic progress, the number of structural types is still far less than the molecules in the molecular world and the nuts and bolts in the macroscopic world. Hence, accumulation of synthetic know-how and mechanistic insights is essential for future development of functional architectures. Going beyond simple structures,^1^ nanohybrids have attracted great attention for the promise of multi-functionality and synergistic effects.^2^

Valence is an important chemistry concept defining an atom's combining power with other atoms. Similar concepts can be defined for satellite nanostructures with a precise number of island domains.^3^ Typically, nanoparticles with a single island are called Janus nanoparticles,^4^ whereas those with more than two islands are called satellite structures. The few examples of satellite structures in the literature were synthesized by polymer attachment,^5^ colloidal assembly,^6^ and growth.^3c,7^ Defining the number of islands in such structures (*i.e.*, achieving valency control) is a critical step towards “total synthesis”^3*a*^ of functional architectures.

**Fig. 1:**
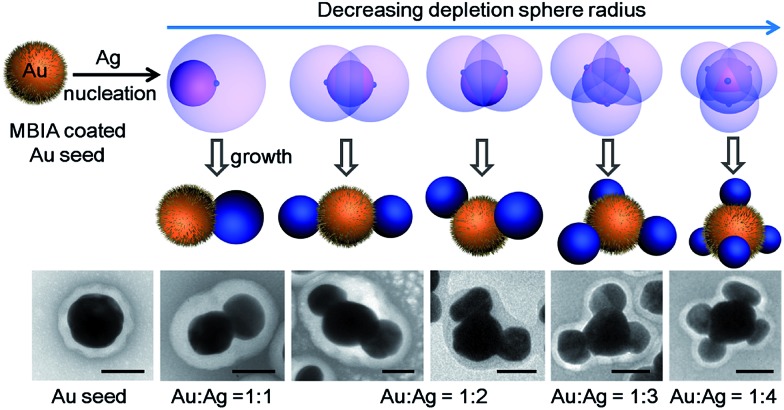
Schematics illustrating the effects of depletion sphere (transparent sphere) in controlling the Ag nucleation on Au seeds and their later growth into Au–Ag Janus/core–satellite structures with different Au : Ag island ratio: 1 : 1; 1 : 2; 1 : 3; and 1 : 4. New nucleation sites can start when the depletion sphere does not fully cover the seed surface. Scale bar: 50 nm.

In this work, we demonstrate the control of valency in the colloidal growth of Au–Ag satellite nanostructures (Fig. 1), where the valency depends on the surface ligand density and the rate of Ag reduction. A depletion sphere mechanism was proposed to explain the choice of nucleation sites as the result of non-steady-state concentration gradient.

## Results and discussion

The multi-island growth was an extension from the previous growth of a single Ag island on a Au seed.^8^ Basically, the ligand 2-mercaptobenzoimidazole-5-carboxylic acid (MBIA) has a –SH group and a diametric –COOH group, allowing it to interact strongly both with the Au core and the subsequent Ag island. Thus, partial embedding of this molecule in between the Au–Ag layers^9^ creates defects and strains, making it possible to tune the Au–Ag interfacial energy.^8,10^ As a result of the “invisible hands of thermodynamics”,^11^ various Au–Ag hybrids ranging from concentric core–shell, eccentric core–shell, acorn, and heterodimer nanostructures have been obtained.

**Fig. 2:**
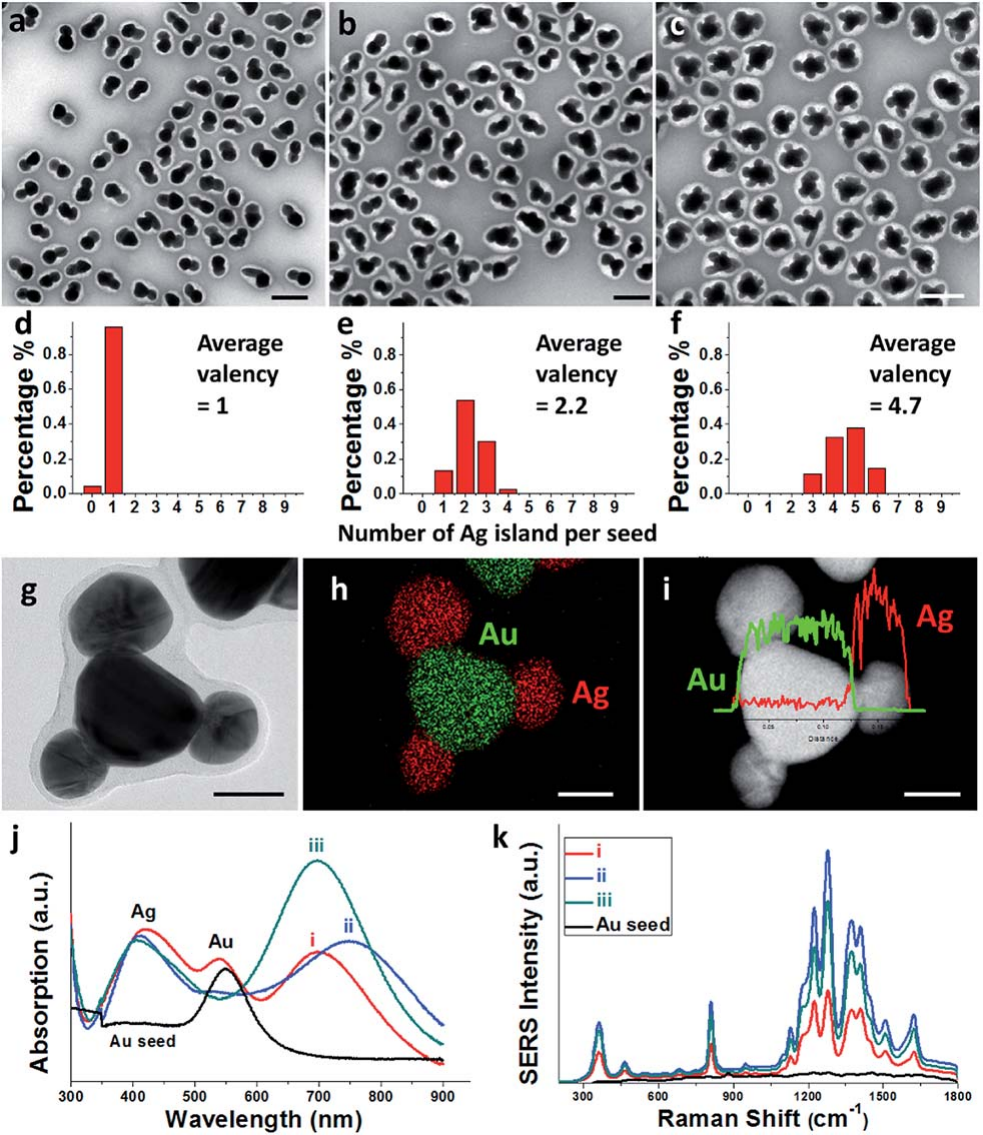
Au–Ag Janus and satellite structures prepared by pre-incubating Au seeds with MBIA ligands at (a) 60; (b) 80; and (c) 100 °C for 2 h; (d–f) histograms showing the distribution of the valency in (a–c); (g–i) TEM image, EDS mapping and line scan of a typical Au(Ag)_3_ satellite nanoparticle; (j–k) UV-Vis absorption and SERS spectra of the nanostructures: curve (i)–(iii) correspond to the samples in (a–c), respectively. Scale bar: 200 nm in (a–c) and 50 nm in (g–i).

We made an interesting observation that improved ligand packing on the seed can lead to a greater number of Ag islands per seed. In a typical synthesis, as-synthesized 70 nm citrate-stabilized Au nanoparticles (Fig. S1†)^8^ were used as seeds. They were incubated with MBIA (20 μM in water) at elevated temperatures (60–100 °C) to allow the ligands to pack well on the nanoparticle surface. After cooling to room temperature, the reductant hydroquinone (HQ) was added to the solution followed by AgNO_3_. With the start of Ag reduction, the resulting Ag atoms were deposited on the seed surface, forming islands. To prevent the product nanoparticles from aggregation, they were encapsulated in polystyrene-*block*-poly(acrylic acid) (PSPAA) shells.^8,12^ As shown previously, the polymer encapsulation was only a method of preservation and did not alter the product structures (Fig. S5†). In the TEM images, the Ag domains often have a lighter contrast than the Au seeds. The polymer shells appear as a white “halo” against the negatively stained background (Fig. 2a–c). With the shells, the nanoparticles are well separated from each other without further aggregation during the drying stage of TEM preparation, making it possible to distinguish the Ag islands. Energy-dispersive X-ray spectroscopy (EDS) mapping and line scans (Fig. 2g–i) verified the Ag islands (red color) on the Au seed (green color).

In contrast to the literature works where Janus/satellite nanostructures were developed in discrete systems, our system can give a range of products by modifying a single factor, for example, the ligand incubation step (Fig. 2). Au–Ag Janus nanoparticles of 95.7% purity were obtained when the Au seeds were incubated with MBIA at 60 °C for 2 h (Fig. 2a).^8^ When this incubation step was carried out at 80 or 100 °C for 2 h, multiple Ag islands were grown on each Au seed, forming satellites. Fig. 2b shows the 80 °C sample with 9.7% Au–Ag dimers, 56% Au(Ag)_2_ trimers, and 35% Au(Ag)_3_ tetramers; Fig. 2c shows the 100 °C sample where most of the seeds have grown 4–6 islands.

Across the samples of Fig. 2a–c, the color changed from brown to dark brown, and then deep green (Fig. S2a and b†), though the total amount of Au and Ag was the same. As shown in Fig. 2j, the UV-Vis spectrum of the Janus nanoparticles (Fig. 2a) showed three peaks at 420, 537, and 700 nm (curve (i) in red color), corresponding to the Ag and Au transverse absorption bands, and the Au–Ag longitudinal absorption, respectively.^8^ In comparison, the presence of Au(Ag)_2_ trimers in Fig. 2b led to a red shift of the longitudinal absorption band to 760 nm (curve (ii) in blue). For the satellite structures as shown in Fig. 2c, the overall round shape caused the longitudinal absorption to shift back to around 700 nm (curve (iii) in dark cyan). This behavior is similar to the multi-layer core–satellite assemblies reported by the Cheng group,^13^ where the broad plasmonic absorption depends on the size and the number of satellite nanoparticles. The SERS signals of the samples roughly followed the same trend. As shown in Fig. 2k, sample 2b with the longest wavelength of the longitudinal absorption has the strongest SERS signal (curve (ii) in blue), likely due to the partial plasmon resonance with 785 nm incident light.^14^

**Fig. 3:**
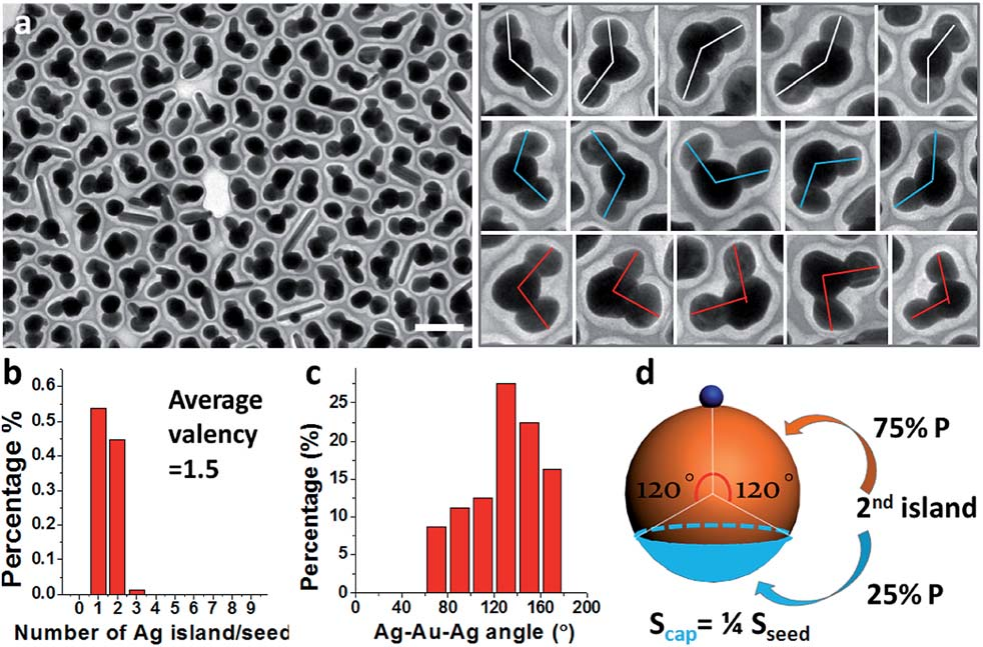
(a) Au–Ag hybrids prepared by pre-incubating Au seeds with MBIA ligands at 60 °C for 15 h; and analyses of the ∠Ag–Au–Ag angles in selected trimers (white color > 120°, cyan ≈ 120°, red < 120°); (b and c) histograms showing the distribution of valency and the ∠Ag–Au–Ag angle; (d) schematics illustrating the probability of second nucleation leading to the observed ∠Ag–Au–Ag angles. Scale bar: 200 nm.

Prolonged incubation with ligands (at a constant temperature of 60 °C) also caused the increase of satellite islands. With the increase of incubation time, the core–satellite structures showed a slight increase of valency (Fig. S6†). At 15 h of incubation, the sample contained 52% Janus nanoparticles and 48% Au(Ag)_2_ trimers (Fig. 3a). Among the trimers, 57% of them were roughly straight (with the Ag–Au–Ag angle ≥ 120°), whereas 43% were more asymmetric with the Ag–Au–Ag angle < 120° (Fig. 3c). Further increase of incubation time to 2 days did not cause obvious increase of valency, suggesting that the effects have reached a plateau.

In these experiments, the incubation step only pre-treats the seeds and all chemical reactions were carried out at room temperature. With the ligand concentration kept the same, it is unlikely that it would affect the degree of Ag^+^ coordination, the rate of Ag reduction, or the rate of dynamic re-adsorption on the freshly generated Ag surface.^15^

It is conceivable that the ligand density on the seed surface would be improved with longer incubation time and higher temperature, both of which can allow the ligand with flat geometry to move around and achieve more orderly packing.^16^ But the two factors are obviously inequivalent in that even the longest incubation time was less effective than the higher temperatures, presumably because some of the packing processes require a higher thermal energy to overcome the kinetic barriers.^16*c*^

**Fig. 4:**
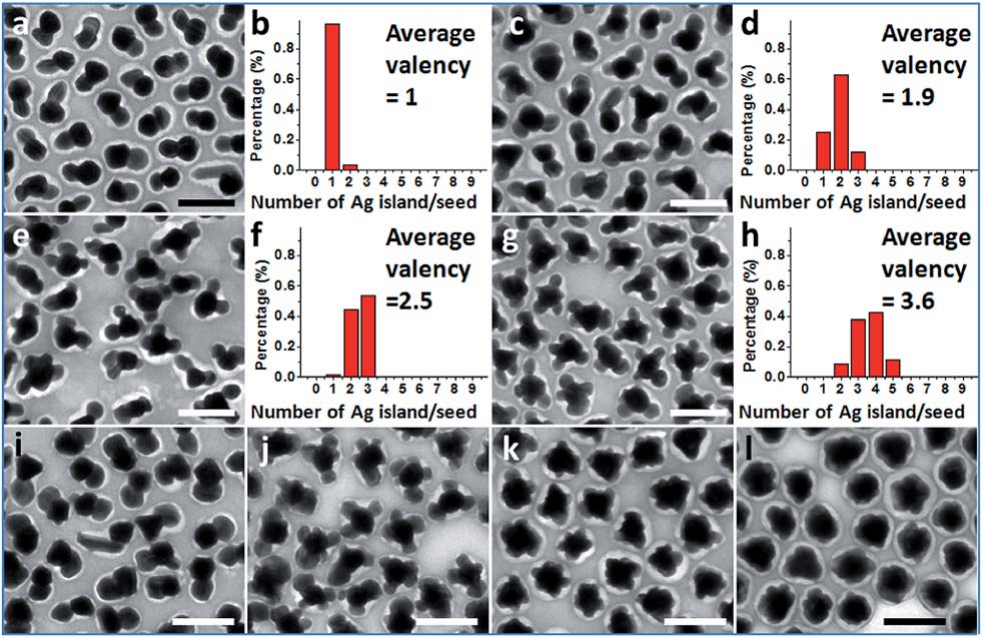
Au–Ag Janus and satellite structures synthesized under different HQ concentration (final): (a and b) 1.6; (c and d) 6.6; (e and f) 13.2; and (g and h) 19.9 mM; (b, d, f and h) histogram showing the distribution of valency; (i–l) Au–Ag Janus and satellite structures synthesized under different NaOH concentration (final): (i) 0.18; (j) 0.36; (k) 0.54; and (l) 0.72 mM. Scale bar: 200 nm.

Further exploration of the preparative conditions revealed that higher concentrations of HQ and NaOH can both promote multi-island growth. In the absence of NaOH and when all other conditions were kept the same, the increase of HQ concentration from 1.6 to 6.6, 13.2, and 19.9 mM caused a monotonous increase in the number of Ag islands per seed (Fig. 4a–h), with the average valency increased from 1 to 1.9, 2.5, and 3.6. Similarly, with HQ concentration (1.1 mM) and all other conditions kept the same, increase of NaOH concentration from 0.18 to 0.36, 0.54, and 0.72 mM led to increase of product valency (Fig. 4i–l). At high NaOH concentrations (0.54 and 0.72 mM), the crowded small Ag islands (>10 per seed) merged with each other to make a continuous shell with a rough surface (Fig. 4k–l). It is apparent that the valency of the satellite structures depends on the rate of Ag reduction. Both the higher reductant concentration and more basic conditions^17^ promote Ag reduction, providing a greater amount of growth materials to the Ag islands and leading to a faster rate of color change. The effects of HQ and NaOH are inequivalent, as even the highest HQ concentration cannot achieve the high valency achieved by the high NaOH concentration (Fig. 4k–l).

**Fig. 5:**
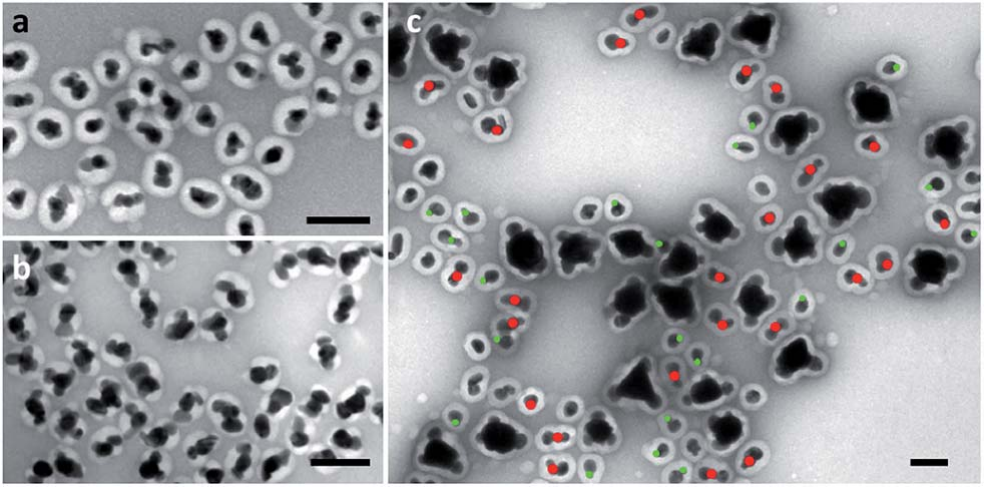
Multi-island growth for (a) 15 nm; and (b) 25 nm seeds. (c) A control experiment where 15, 25, and 70 nm seeds are mixed to achieve the same reaction conditions. Green and red dots were used to distinguish the 15 and 25 nm seeds, respectively. Scale bar: 100 nm.

The multi-island growth can be achieved on seeds of various sizes. In the above discussion, 70 nm seeds are chosen to demonstrate a complete range of valency. As shown in Fig. 5a and b, the 15 and 25 nm Au seeds can also lead to a multi-island structure with modified reaction conditions.^18^ A significantly higher NaOH concentration was necessary for the small 15 nm seeds. We expect that the average valency should depend on the relative size of the seeds under the same reaction condition. To test this expectation, a control experiment was carried out using pre-mixed seeds of 15, 25 and 70 nm, giving average valencies of 1.1, 1.6 and 3.8, respectively (Fig. 5c). It should be noted that the size of the Ag islands on all seeds was similar, indicating that the initial Ag nucleation occurred roughly at the same time.

It appears that the Ag islands form very quickly in the reaction. Fig. S7† shows the kinetic UV-Vis absorption spectra during the growth of the sample in Fig. 3a. The appearance of Ag transverse absorption at 410 nm and the longitudinal absorption at >750 nm indicates that significant Ag islands have already formed within the first scan (<1 min) and that the islands were fully grown by 5 min. It is conceivable that the initial island formation should occur much earlier than any Ag island of plasmonic significance.

Previously, the mechanisms of Janus nanoparticles in the literature focused on the symmetry-breaking growth behavior—why an island is formed instead of a core–shell structure. In this branch of mechanistic discussion, symmetry-breaking can be achieved when: (1) part of the seed is covered by a special capping ligand^5a,19^ or polymer coating;^20^ (2) growth is preferred at the defect sites, such as stacking faults, twin plane, or dislocation;^7c,21^ (3) primary seed particles aggregate with a special manner followed by oriented attachment;^22^ or (4) various types of kinetic control, including supply of growth material *via* reaction rates,^23^ and ligand kinetics.^15^ We have discussed the detailed thermodynamic and kinetic arguments in our previous works.^8,11,15^

In this work, we focus the discussion on the next step—why only one island is grown and what are the underlying reasons for the lack of islands on the remaining seed surface. In the literature, control of reactant concentrations or their rates of addition is a common method to exert synthetic control. The resulting satellite structures^7b,24^ or partially encapsulated structures^23b,g^ were often broadly explained by “kinetic control”, but there is a lack of theory to explain the detailed steps from the rate of reactions to the shape of nanostructures. Our ability in controlling the “on/off” of multi-island growth permits new mechanistic enquiries. We believe that further exploration of the detailed mechanistic scenario (as opposed to identifying a single factor) would greatly enhance our understanding and permit new synthetic designs.

**Fig. 6:**
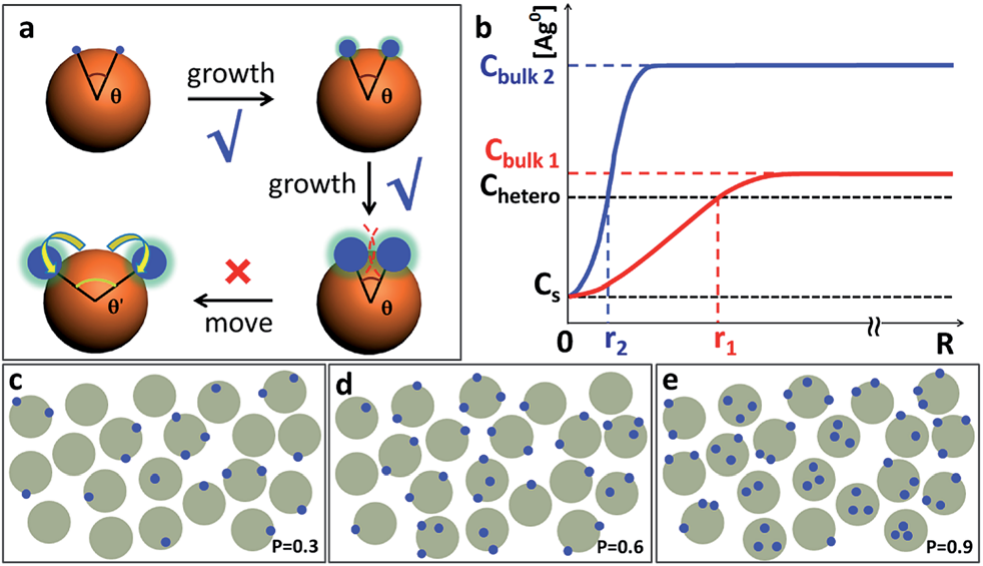
(a) Schematics highlighting the immobility of the Ag domains. The critical point of choice occurs when the domains were ultrasmall and then, the ∠Ag–Au–Ag angle can no longer change; (b) plot of Ag^0^ concentration *vs.* radius: the red and blue curves represent low and high initial oversaturation (*C*_bulk_), respectively. *r*_1_ and *r*_2_ are the radius of depletion sphere in nucleation stage under the corresponding *C*_bulk_; (c–e) schematics illustrating the distribution of nucleation sites (blue dots) under random nucleation, after 3 rounds of nucleation with (c) *P* = 0.3; (d) *P* = 0.6; and (e) *P* = 0.9. As Janus nanoparticles build up in concentration, random nucleation would start to make Au(Ag)_2_ trimers, so on and so forth. It would be impossible to achieve high-purity Au–Ag Janus nanoparticles regardless of the *P* value.

Throughout the images in Fig. 2–4, the Ag islands on any Au seed are well separated from each other, as if there is repulsion among them. Intuitively, one may invoke steric or charge repulsion. However, the Ag islands are neither liquid nor incoming particles,^6*a*^ incapable of moving to a different location under the influence of repulsion (Fig. 6a). At the point of initiation, the Ag islands must be ultrasmall and the repulsion among them needs to be extraordinary to exert sufficient influence. In the literature, polymer nanodroplets can grow on the surface of a particle or substrate, forming symmetrical satellite islands with similar appearance.^5*c*^ While it is possible that precursor droplets or highly swollen polymer domains may adjust their locations, it is impossible for the Ag islands here.

Should the nucleation occur randomly on the Au surface, the probability of nucleation should be proportional to the surface area. For a Janus nanoparticle (Fig. 2a), at the point of initiation, the ultra-small Ag nucleus should occupy less than 10% of the nanoparticle surface. If we set the probability of the initial nucleation as *P*, the probability of a seed with second nucleation should be >*P* × 0.9*P*. No matter what the *P* value is, it is virtually impossible to achieve one Ag island per seed (with eventual 95.7% probability) and then stop there (Fig. 6c–e). Thus, the absence of high-valency products in Fig. 2a is strong evidence against the notion of random nucleation. For growing the second Ag island and making Au(Ag)_2_ trimers, it appears more probable (Fig. 3d) to achieve the ∠Ag–Au–Ag angle < 120° (75% probability) than >120° (25%). This is proven wrong by the experiments, where the latter structure (≥120°) was adopted by over 57% of the Au(Ag)_2_ products (Fig. 3a and c).

For the above theories of “repulsion” and “random nucleation”, it is critical to find the detailed steps linking those mechanisms to the observations. For example, how exactly are the different degrees of repulsion affected by the surface ligand density or the rate of Ag reduction; and how those factors can control the valency of the final product. However, no plausible links could be found. After repeated experiments, we realized that both the surface ligand density and the rate of Ag reduction would lead to increased oversaturation during the initial nucleation stage, thus providing a possible link to a plausible mechanism.

Specifically, a dense layer of ligand would force the reduced Ag atoms to build up in the solution. With ligands nearly covering all seed surfaces, the increased surface energy^11^ is manifested by the gradual decrease of “wetting” of Ag domains on Au,^8^ and this would increase the cost of initial nucleation.^25^ On the other hand, a faster rate of Ag reduction would supply more growth material to initiate and sustain the higher oversaturation. These arguments explain well the lack of multi-island growth in the presence of no/low ligand concentrations (giving core–shell structures),^8^ as the rapid growth on the seeds would consume the growth material (Ag atoms) preventing their build-up.

**Fig. 7:**
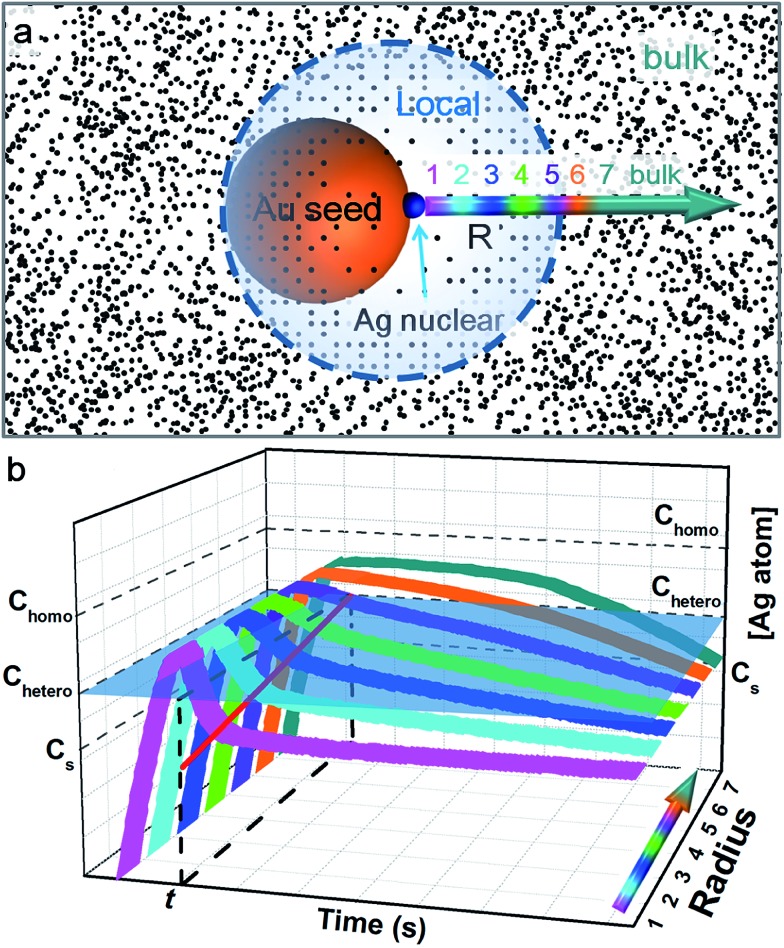
(a) Schematics illustrating the depletion sphere model, where a concentration gradient (represented by the black dots and shown as the color gradient) exists around the growth site (blue dot) and a spherical boundary marks the threshold concentration *C*_hetero_; (b) plot of Ag^0^ concentration *vs.* time and location. The different color marks the locations as indicated in a. The initial stage of Ag^0^ build-up is the same for all locations. Once a nucleation site initiates, its vicinity (pink) is depleted faster than the locations further away, generating a concentration gradient (the red line on the vertical plane). Within the depletion sphere, the Ag^0^ concentration is below *C*_hetero_ (the grey transparent plane), whereas it is above the plane outside the depletion sphere, but still below *C*_homo_. In other words, heterogeneous nucleation can occur outside the sphere (requires seed surface) but no homogeneous nucleation.

In order for heterogeneous nucleation to occur on the ligand-covered Au seed, the oversaturation of the Ag^0^ in the solution must surpass a certain threshold (*C*_hetero_),^25^ which is in general lower than the threshold for homogeneous nucleation (*C*_homo_, Fig. 7). Basically, the random collision of Ag^0^ atoms must reach a nucleus of critical size. With partial bonding with the seed surface, the critical nucleus can be smaller on the seed than that formed in middle of the solution.^11^ On these bases, we speculate that a new island cannot occur too closely to an old island because the growth at the existing site would deplete the growth materials nearby, prohibiting new nucleation sites.

Previously, the concept of a “depleted region” was invoked to explain the controlled growth of CaCO_3_ crystals on a flat substrate with patterned self-assembled monolayer (SAM).^26^ At steady-state, the depleted radius was estimated to be about 80–100 μm, about 4 orders of magnitude larger than the short-distance (10–20 nm) nucleation in this work. In comparison, the Ag deposition on the colloidal Au seeds occurs in a 3-dimensional space, and the seeds undergo constant Brownian motion. The seed concentration is estimated to be 5.66 × 10^–11^ M, that is, each seed occupies an average space of about 3 × 3 × 3 μm^3^. The fact that pure Ag nanoparticles were not formed suggested that, at some point, the depleted region (with *C*_homo_ as boundary) must have extended over the average space and inhibited homogeneous nucleation. This, however, cannot explain the control of heterogeneous nucleation at short distance. It is also clear that the depleted region at such a large radius must be severely disrupted by the rapid Brownian motion of the seeds.

Hence, we propose a depletion sphere model to explain the effects of a non-steady-state concentration gradient at the nanometer range, which occurs faster than the time-scale of Brownian motion. More specifically, the initial nucleation on a seed and the subsequent growth are expected to partially deplete the local Ag^0^ atoms, leading to a concentration gradient (Fig. 7a). The chemical reaction producing the Ag^0^ atoms should occur homogeneously throughout the solution (3 × 3 × 3 μm^3^ per seed), but their depletion only occurs at the growth site on the seeds (70 nm). Thus, the chemical reaction at the vicinity of the seed is clearly insufficient to sustain the Ag growth. With the concentration gradient, a spherical boundary can be defined as the depletion sphere, within which the oversaturation is too low (<*C*_hetero_) to achieve heterogeneous nucleation. Thus, if the depletion sphere does not fully cover the seed surface, a second nucleation site could occur, and the probability should depend on the exposed area and the degree of oversaturation (Fig. 1). The size of the depletion sphere relative to the size of seed (Fig. 5) determines the number of nucleation sites, and thus, the number of islands after the growth.

Once a Ag nucleus forms on the seed surface, the absence of ligands on the “fresh” surface would make it greatly more favorable for Ag^0^ deposition than the “old” seed surface with dense ligands.^8^ As previously discussed, the dynamic ligand re-adsorption has to compete with the Ag deposition in order to inhibit the “fresh” surface.^15^ Hence, the “ligand inhibition” in this work refers to a dynamic and competitive inhibition, rather than a static and absolute inhibition.

In an ideal model, efficient growth at the Ag island would deplete the Ag^0^ atoms at their immediate vicinity, reaching saturation (*C*_s_), *i.e.*, it is a perfect “sink” and the diffusion does not cause build up of Ag^0^. The depleted region rapidly expands, creating a concentration gradient at the radial direction. The diffusion can be described by the classic Fick's laws,^27^ where the change of concentration with time is proportional to the second derivative of the concentration (eqn (1)) and *D* is the diffusion coefficient.1
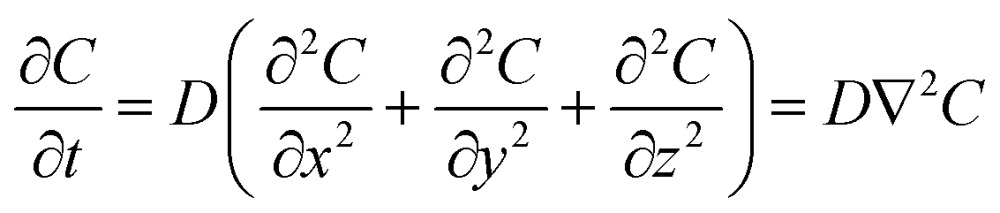

2
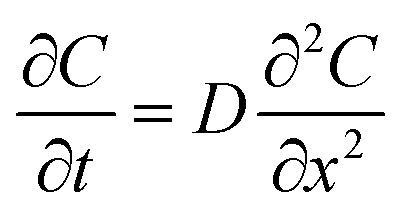



To simplify, eqn (1) can be expressed at the radial direction as eqn (2). In an ideal model, we can set a constant *C*_bulk_. Solving the equation leads to the Gauss error function with a sigmoid shape concentration gradient (Fig. 6b), which depends on the time and boundary conditions. A few basic conclusions can be drawn: (1) the depletion sphere as defined by the concentration gradient would expand with time; (2) at the steady state (∂*C*/∂*t* = 0), a constant gradient would be established from the origin to the bulk solution; (3) the higher the initial oversaturation is, the steeper the concentration gradient becomes, and thus, the depletion sphere defined by *C*_hetero_ is smaller in radius (Fig. 6b, *C*_bulk 2_ > *C*_bulk 1_, thus *r*_2_ < *r*_1_).

This simplified model of diffusion has to unite with the classic model of nucleation and growth, to reflect the drastic change of concentration during the critical stage of the initial nucleation (*i.e.*, the non-steady-state concentration gradient). Fig. 7b depicts the concentration trace across the radius: before heterogeneous nucleation, the Ag^0^ concentration at all locations is forced to build up, surpassing *C*_hetero_. After nucleation, the vicinity (location 1) is most rapidly depleted and the farther locations are gradually depleted due to the diffusion across the concentration gradient. Only the locations 6 and 7 outside the depletion sphere are able to maintain a higher concentration above *C*_hetero_ for a sizable period.

In order to explain the inhibition at the nanometer range, the critical point of choice should occur during the initial expansion of depletion sphere, rather than after establishing steady-state concentration gradients (slower than the time-scale of Brownian motion). At the outskirts of the depletion sphere, the probability of the second nucleation is not a constant. It has to be “integrated”, taking into consideration both the changing size of the “exposed” area and the changing degrees of oversaturation (Fig. 7b). The probability of nucleation should increase with the degree of oversaturation (at some point it would approach 1), but the detailed dependence is still unknown in the literature. It should be also noted that *C*_bulk_ is affected not only by diffusion (depletion) but also by the chemical reaction (supply) which varies with time.

For making Janus nanoparticles, the lower oversaturation means that the depletion sphere of the first nucleation has time to expand given the lower probability of the second nucleation. For multi-island (>3) satellite structures, there is a sequence of nucleation sites and depletion spheres. The steeper concentration gradient and higher nucleation probability would cause larger variance in terms of total nucleation sites per seed. This explains the broadening distribution of island number in Fig. 2a–f and 4a–h.

## Conclusions

With this growth/diffusion model, we provide detailed steps linking the initial conditions (incubation time and rate of reduction) to the valency of the resulting nanostructures: both the higher ligand density on the seed and faster Ag reduction rate lead to higher initial Ag^0^ oversaturation in the bulk (*C*_bulk_), which in turn leads to a steeper concentration gradient and higher probability of nucleation. The resulting smaller depletion sphere thus leads to more Ag islands (Fig. 1). Most importantly, the inhibitive role of the depletion sphere can be broadly applied to explain the lack of new nucleation sites during the growth of Janus nanoparticles (*i.e.*, the resulting high purity) and the mysterious spacing among the islands of satellite nanostructures.

It is conceivable that all nucleation processes would deplete their immediate vicinity and inhibit new nucleation within the radius. Recognizing this critical inhibitive role is, in our opinion, essential for rational design of colloidal syntheses. Rather than identifying a critical growth factor, we endeavour to give a complete mechanistic scenario consistent with fundamental growth principles. Admittedly, this opens up a broad frontier, many aspects of which are still unknown in the literature. Nevertheless, we believe that the new synthetic know-how and the mechanistic insights in this work would contribute to the advance of nanosynthesis.
