# Supplementary material for: Depletion sphere: Explaining the number of Ag islands on Au nanoparticles
Source: Chem Sci. 2016 Aug 17;8(1):430–6. doi: 10.1039/c6sc02276f (PMC5365066; doi:10.1039/c6sc02276f)
Supplement: Supplementary file 1 [file SC-008-C6SC02276F-s001.pdf]

Supporting Information

Depletion Sphere: Explaining the Number of Ag Islands on Au Nanoparticles

Yuhua Feng, Yawen Wang, Xiaohui Song, Shuangxi Xing,* and Hongyu Chen*

Email: iashychen@njtech.edu.cn; xingsx737@nenu.edu.cn;

Materials and Instruments: All chemicals were used as received without further purification. Hydrogen tetrachloroaurate(III) hydrate ($HAuCl_4 \cdot 3H_2O$), 99.9% (metal basis Au 49%) was purchased from Alfa Aesar; sodium citrate tribasic dihydrate (99.0%), hydroquinone (99.5%) and $AgNO_3$ (99.9999%) were purchased from Sigma-Aldrich; 2-mercaptobenzoimidazole-5-carboxylic acid was purchased from Tokyo Chemical Industry Co., Ltd. (TCI); 1,2-dipalmitoyl-sn-glycero-3-phosphothioethanol (Sodium salt, abbreviated as PSH) was purchased from Avanti Polar Lipids, Inc.; amphiphilic diblock copolymer polystyrene-*block*-poly(acrylic acid) (PS_{154}-*b*-PAA_{49}, M_n = 16000 for the polystyrene block and M_n = 3500 for the poly(acrylic acid) block, M_w/M_n = 1.15) was purchased from Polymer Source, Inc.; Deionized water (resistance > 18.2 MΩ·cm) was used in all reactions. Copper specimen grids (300 mesh) with formvar/carbon support film (referred to as TEM grids in the text) were purchased from Beijing XXBR Technology Co.

TEM images were collected by using a JEM-1400 (JEOL) Transmission Electron Microscopy operated at 100 kV. $(NH_4)_6Mo_7O_{24}$ was used as stain, against which the polymer shells appear white color. Energy-dispersive X-ray spectroscopy (EDS) mapping and line scan was carried out on a JEOL 2100 F Field Emission Transmission Electron Microscope at 200 kV. UV-Vis spectra were collected on a Cary 100 UV-Vis spectrophotometer (VARIAN, as-synthesized colloid was used without further purification). Raman spectra of the sample solution were collected on a PeakSeeker Pro spectrometer (Raman Systems Inc.) in a 4mL glass vial (15×45mm) under a red laser (λ = 785 nm) at 290 mW (direct measurement of the colloid).

Synthesis of Au seed nanoparticles. 15 and 25 nm Au nanoparticles were synthesized according to the method reported by Frens.[1] 70 nm Au nanoparticle was synthesized according to our previous work.[2]

Synthesis of Au-Ag Janus nanoparticles. To 1 mL as-synthesized citrate-stabilized 70 nm Au nanoparticles,[24] 2-mercaptobenzoimidazole-5-carboxylic acid (MBIA, in ethanol) was added (final 0.02 mM) under vigorous vortex. The mixture was incubated in a 60 °C oven for 2 h. After the solution was cooled down to room temperature, hydroquinone (1.1 mM) and $AgNO_3$ (0.53 mM) were added in sequence under vigorous vortex. The resulting solution was placed undisturbed at room temperature for at least 2h to allow the complete reduction of $AgNO_3$.

Synthesis of Au-(Ag)$_n$ satellite structures by using 70 nm Au seeds. The Au-(Ag)$_n$ core-satellite nanostructures were prepared by following the same procedure as stated in the above synthesis, with only one factor varied, for example, the incubation temperature was increased from 60 to 80 and 100 °C (Fig. 2b-c); or the incubation time was prolonged to 15h (Fig. 2g); or change HQ concentration (Fig. 3a-h); or add different amount of NaOH before the addition of HQ and $AgNO_3$ (Fig. 3i-l). All the resulting nanostructures were encapsulated inside PSPAA polymer shell for TEM measurement.

Synthesis of Au-(Ag)$_n$ satellite structures by using small Au seeds. The synthesis of Au-(Ag)$_n$ core-satellite nanostructures with small Au seed nanoparticles followed the same procedure of that for 70nm Au seeds with slight modification. In details, the [MBIA] in pre-incubation is 20 µM in both cases, other conditions are: $[NaOH]_{final}$ = 0.88 mM, $[HQ]_{final}$ = 1.07 mM, $[AgNO_3]_{final}$.= 0.53 mM for 15 nm Au seeds and $[NaOH]_{final}$ = 0.27 mM, $[HQ]_{final}$ = 0.74 mM, $[AgNO_3]_{final}$.= 0.37 mM for 25 nm Au seeds.

Synthesis of Au-(Ag)$_n$ satellite structures by using mixed Au seeds. Firstly, 1 mL seed solution was prepared by mixing 15, 25 and 70 nm Au nanoparticles in 2:1:1 volume ratio, then following a same procedure stated above for the synthesis of Au-(Ag)$_n$ core-satellite nanostructures, detailed conditions are: [MBIA] in pre-incubation is 20 µM, $[NaOH]_{final}$ = 0.31 mM, $[HQ]_{final}$ = 1.35 mM, $[AgNO_3]_{final}$.= 0.68 mM.

Polymer encapsulation of the nanostructures. The as-synthesized sample solutions were used directly in the encapsulation step. In details, 200 µL of the resulting nanostructure solution were added into 880 µL DMF under vortex, followed by the addition of PSPAA (80 µL, 8 mg /mL in DMF) and PSH (40 µL, 2 mg /mL in EtOH). The mixture was heated in a 110 °C oil bath for 2 h to induce the polymer self-assembly. After cooling down to room temperature, the mixture was diluted by water to de-swell and immobilize the polymer shells, the resulting nanoparticles were isolated by centrifugation for TEM measurements.

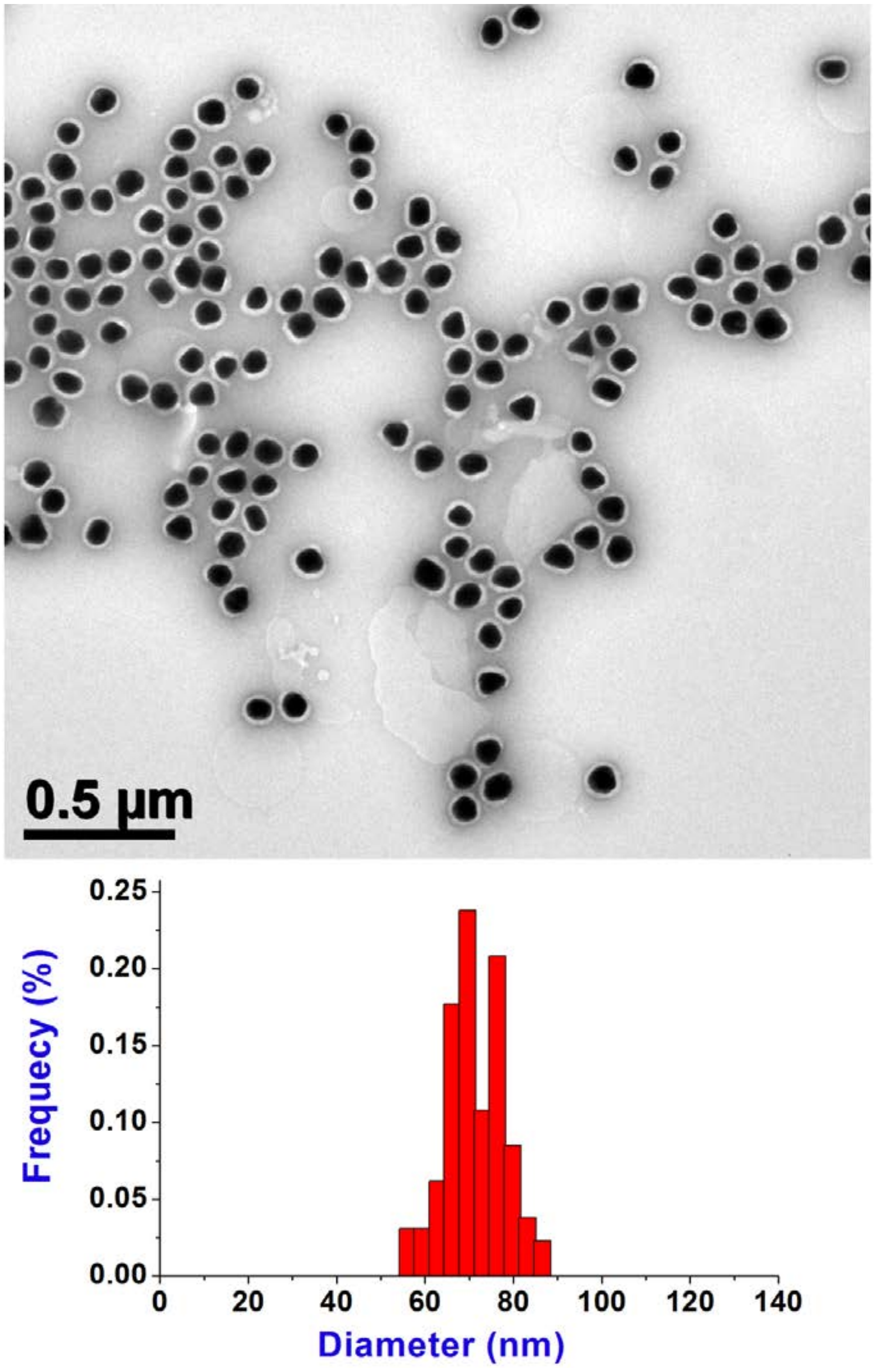

Fig. S1 TEM image and size distribution of 70 nm Au seeds encapsulated in PSPAA shell. The PSPAA shell can protect the nanoparticles from aggregation and the morphology details can be observed more clearly.

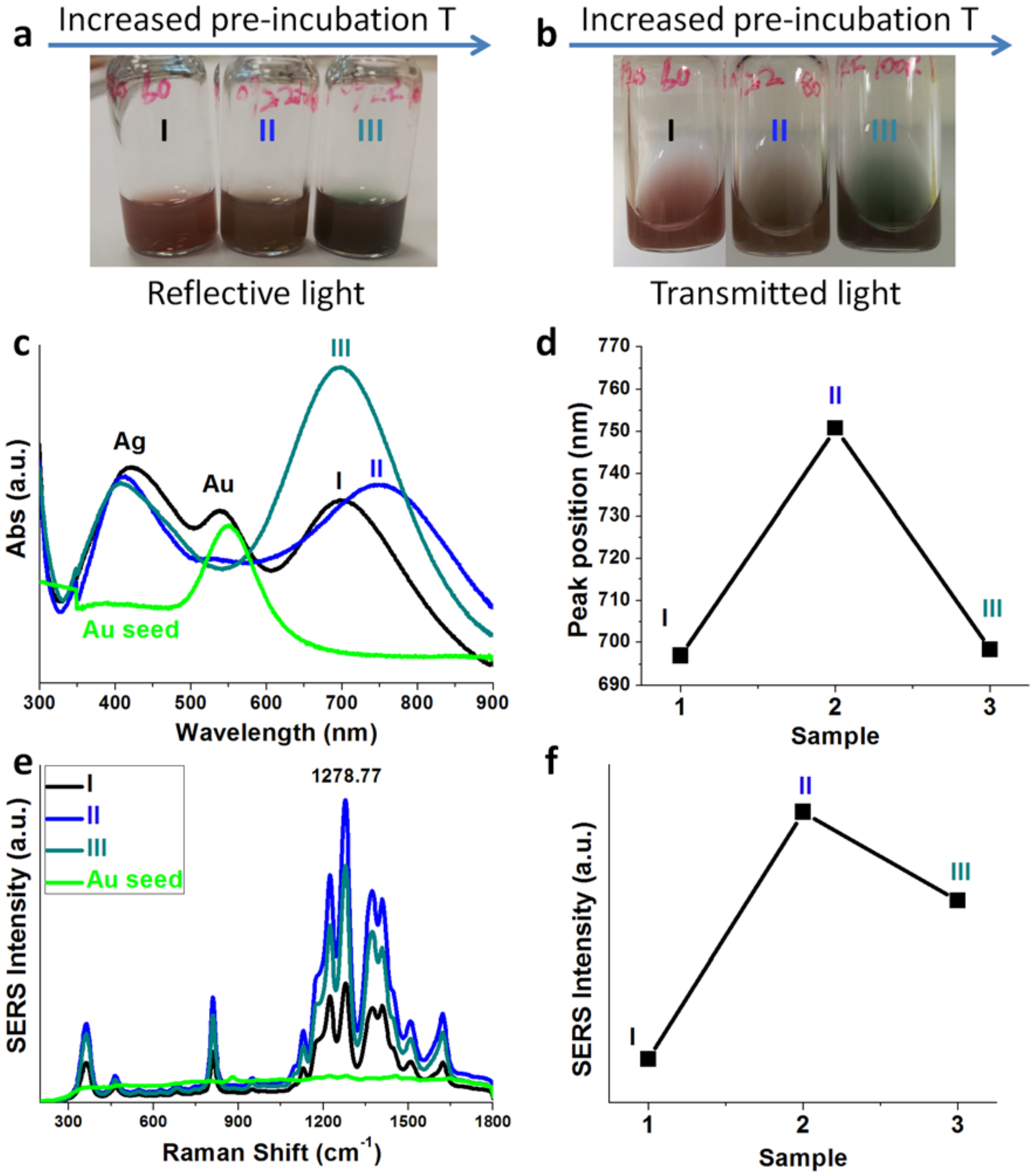

Fig. S2 (a, b) Photograph of the samples in Fig. 2 (I-III correspond to the samples in Fig. 2a-c), which showed different color under both transmitted and reflected light; (c, d) UV-vis absorption spectra and the plot indicating the peak position of the longitudinal absorption and (e, f) SERS spectra and the plot indicating the intensity of the Raman peak at 1278.77 cm^{-1} of the three samples.

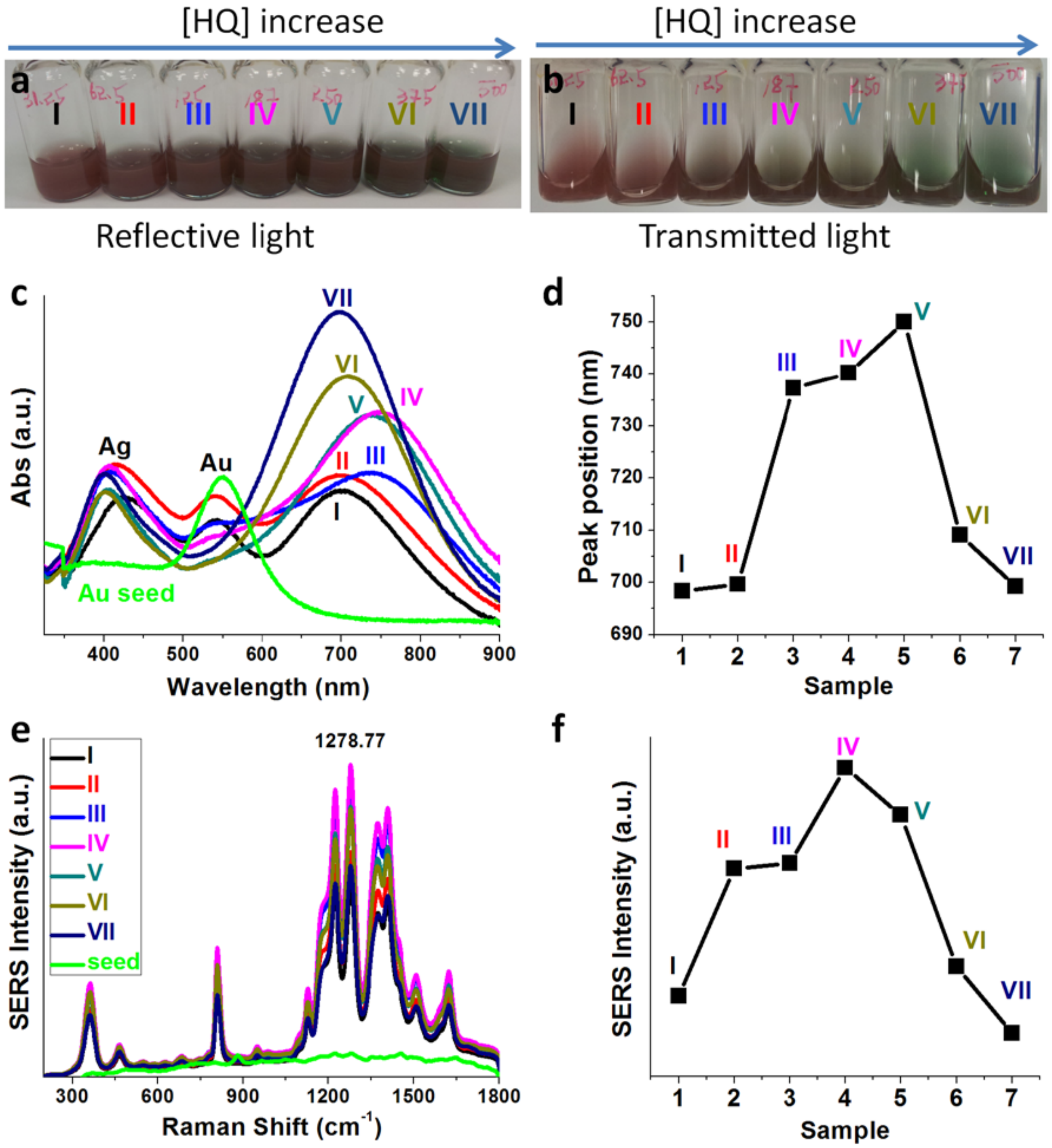

Fig. S3 (a, b) Photograph of the samples synthesized under different final HQ concentration of (I) 1.64, (II) 3.28, (III) 6.56, (IV) 9.94, (V)13.25, (VI) 19.88, and (VII) 26.3 mM (final), which showed different colors under both transmitted and reflected light; (c, d) UV-vis absorption spectra and the plot indicating the peak position of the longitudinal absorption and (e, f) SERS spectra and the plot indicating the intensity of the Raman peak at 1278.77 cm^{-1} for the different samples.

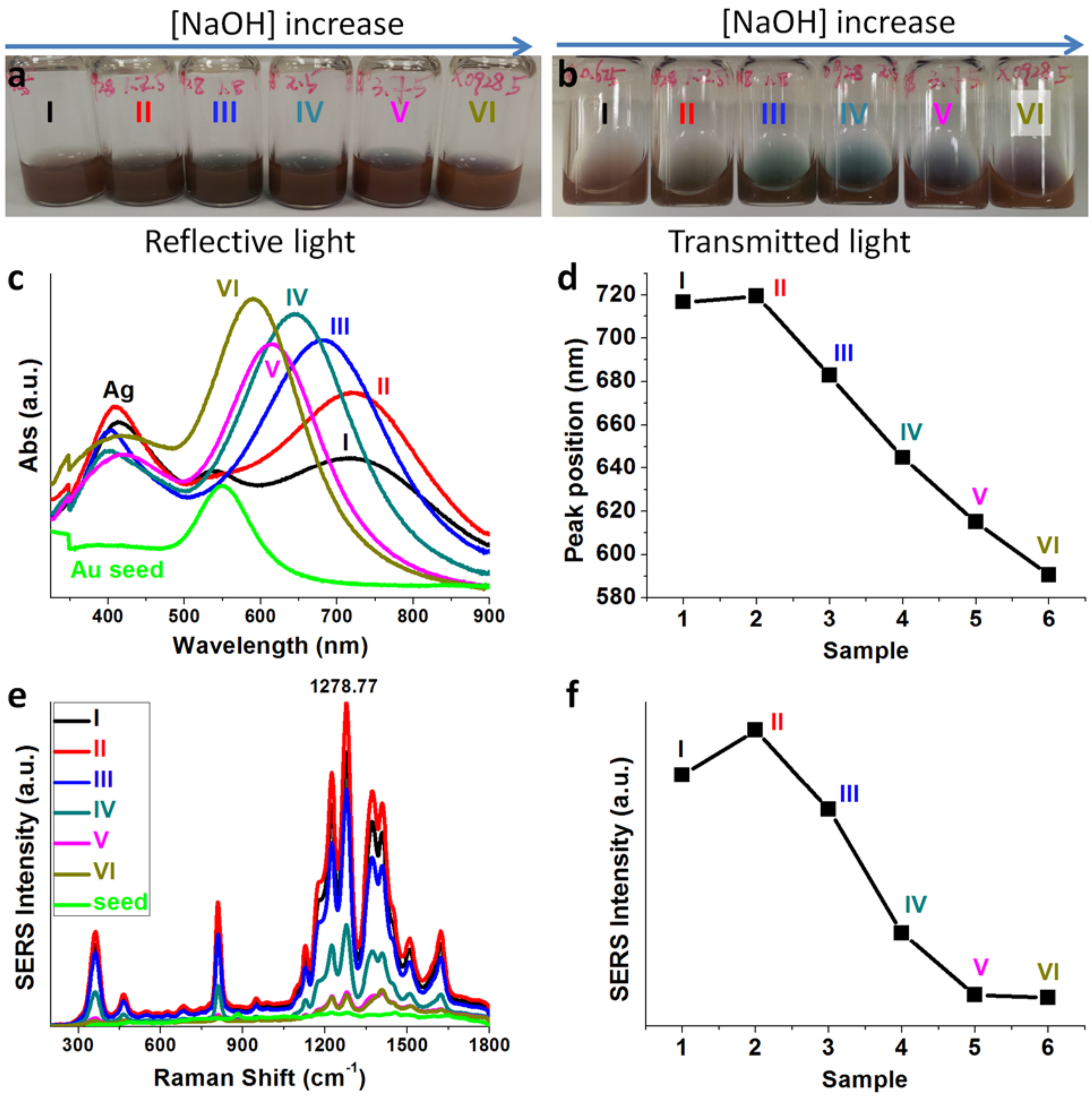

Fig. S4 (a, b) Photograph of the samples synthesized under different NaOH concentration of: (I) 0.09, (II) 0.18, (III) 0.26, (IV) 0.36, (V) 0.54, and (VI) 0.72 mM (final), which showed different colors under both transmitted and reflected light; (c, d) UV-vis absorption spectra and the plot indicating the peak position of the longitudinal absorption and (e, f) SERS spectra and the plot indicating the intensity of the Raman peak at 1278.77 cm^{-1} for the different samples.

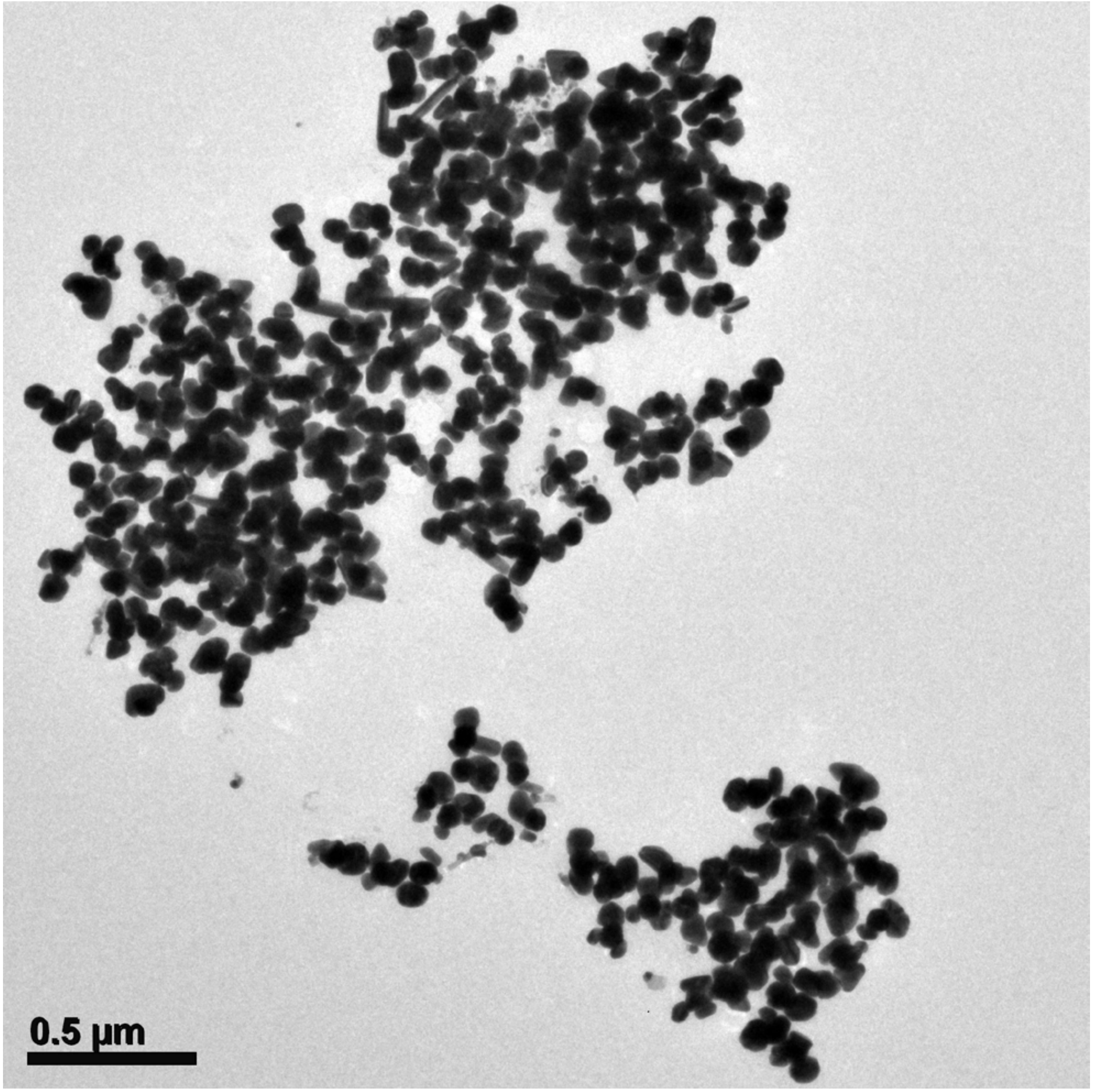

Fig. S5-1 Large-view TEM image of the Au-Ag satellite nanoparticles before PSPAA encapsulation (no obvious change in structure can be observed before and after encapsulation of PSPAA polymer shell), the same sample as shown in Fig. 2b.

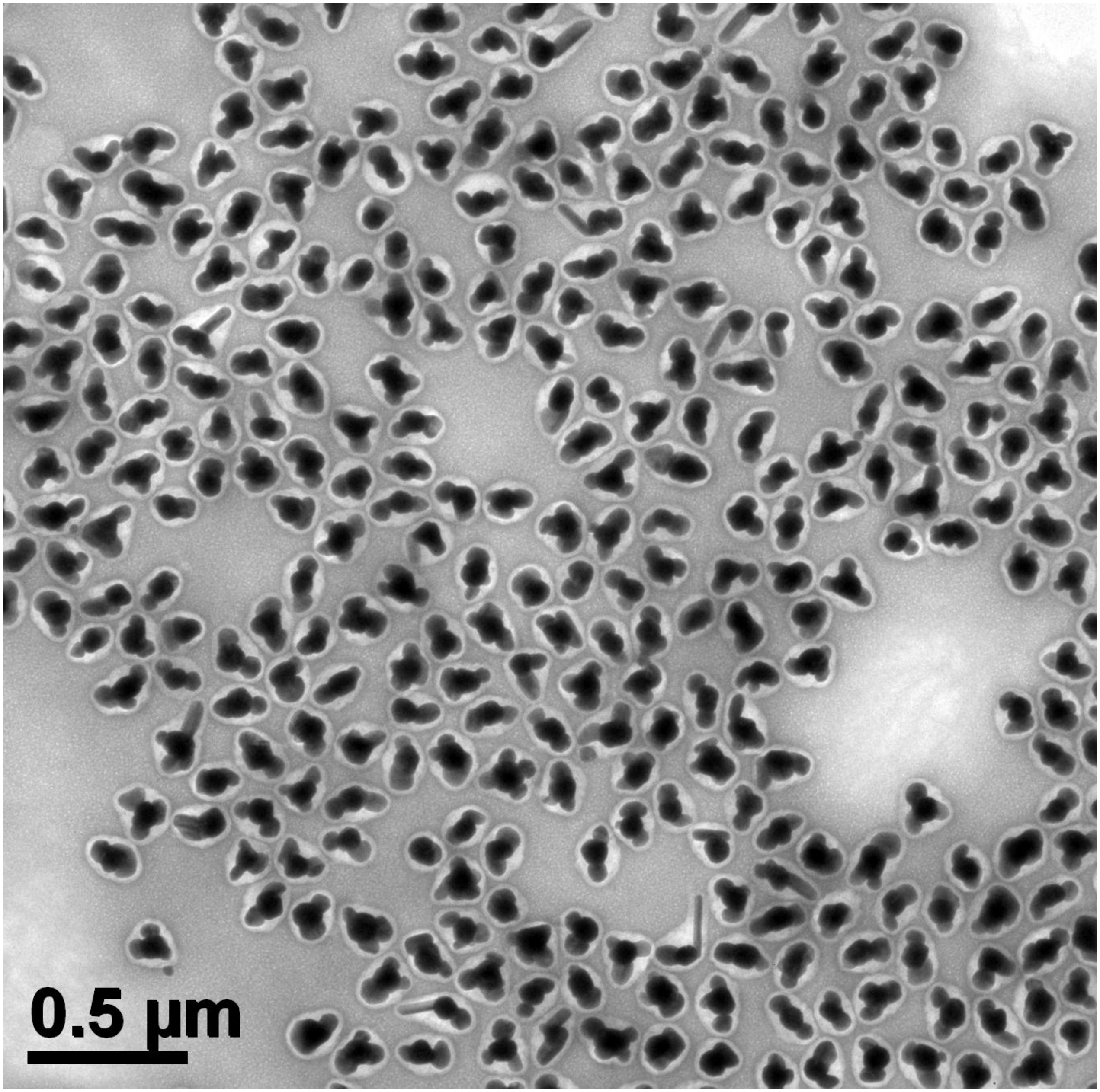

Fig. S5-2 Large-view TEM image of the Au-Ag satellite nanoparticles before after PSPAA encapsulation (no obvious change in structure can be observed before and after encapsulation of PSPAA polymer shell), the same sample as shown in Fig. 2b.

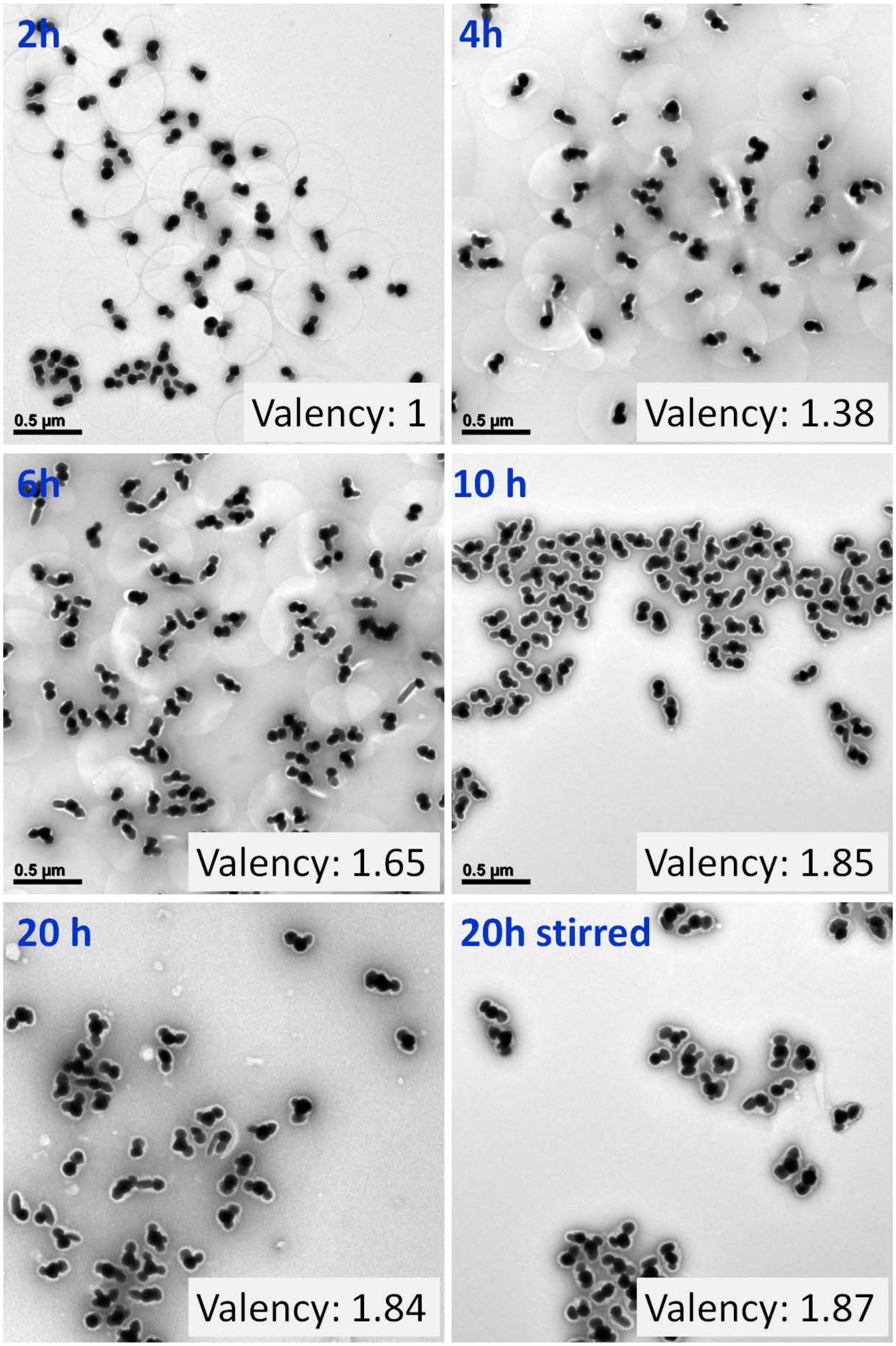

Fig. S6 TEM images of Au-Ag core-satellite structures obtained under different pre-incubation time (2-20 h). The valency of Au seeds increased with increasing incubation time. When the incubation time is longer than 10 h, the valency will not change obviously, suggesting that the incubation time effects have reached a plateau. Note: the growth condition is the same with that in Fig. 3 in the main text, except for the incubation time. Similar valency of $AuAg_n$ satellites nanostructures (20 h incubation) was observed for that with/without vigorous stirring (the maximum speed of 1400 rpm) during the reaction.

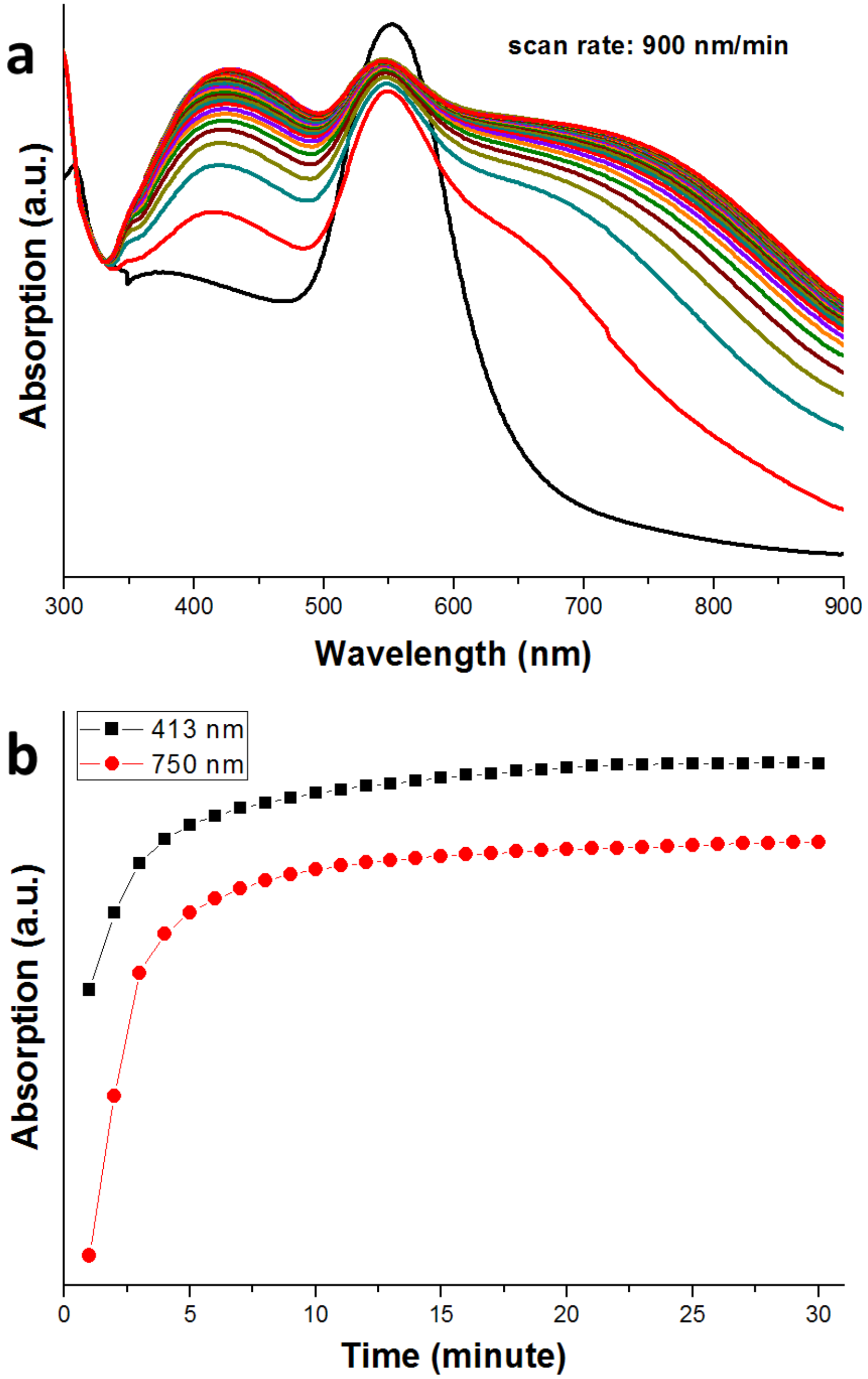

Fig. S7 (a) UV-Vis kinetics and (b) plot of absorption against the reaction time for the peak at 413 nm (red color, Ag transverse absorption) and 750 nm (black color, longitudinal absorption) during the growth of Ag islands on Au seeds under the same experiment condition as that in Fig. 3 in the main text.

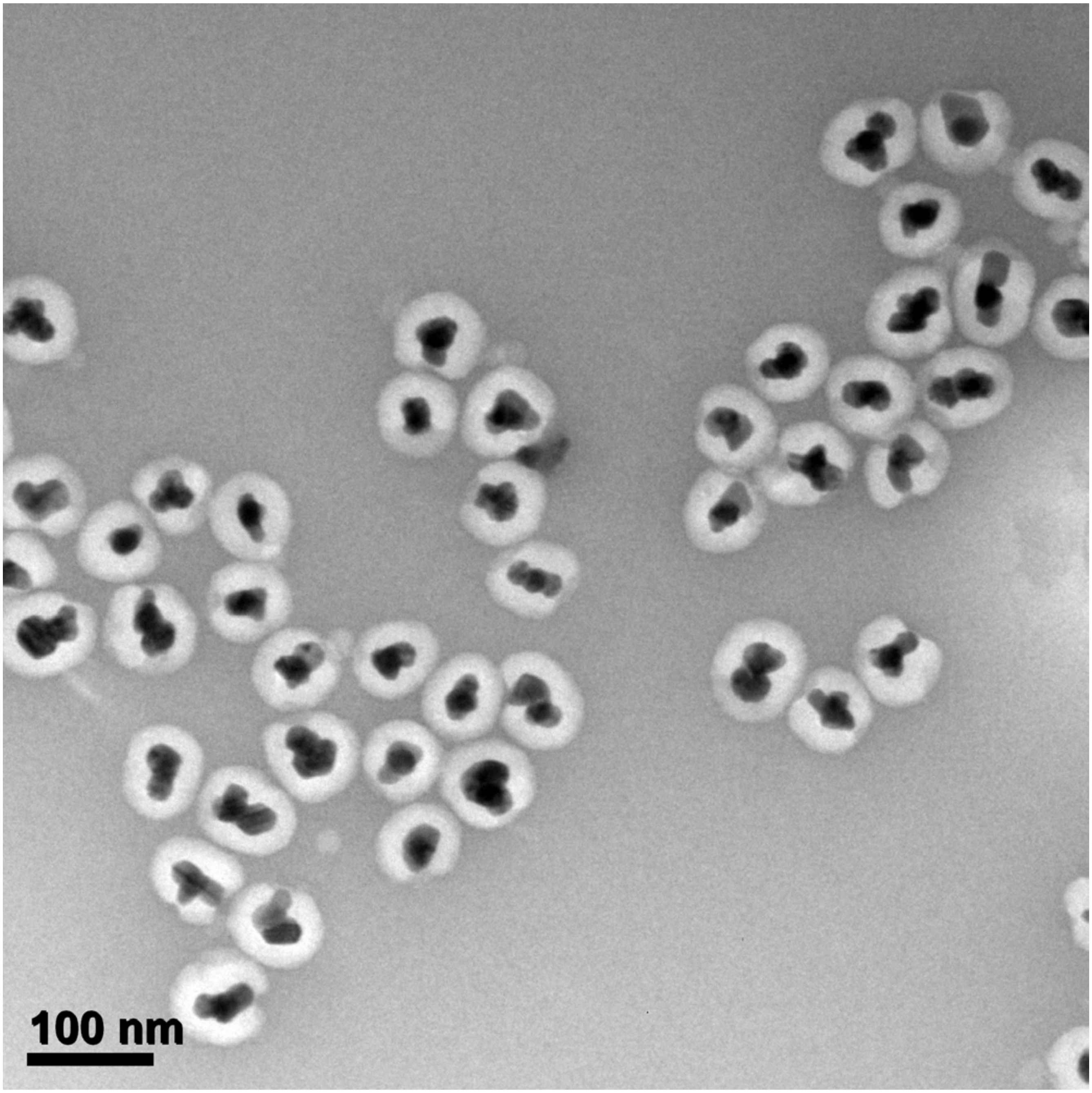

Fig. S8-1 Large-view TEM image of the Au-Ag_n satellite structures, the same sample as shown in Fig. 5a.

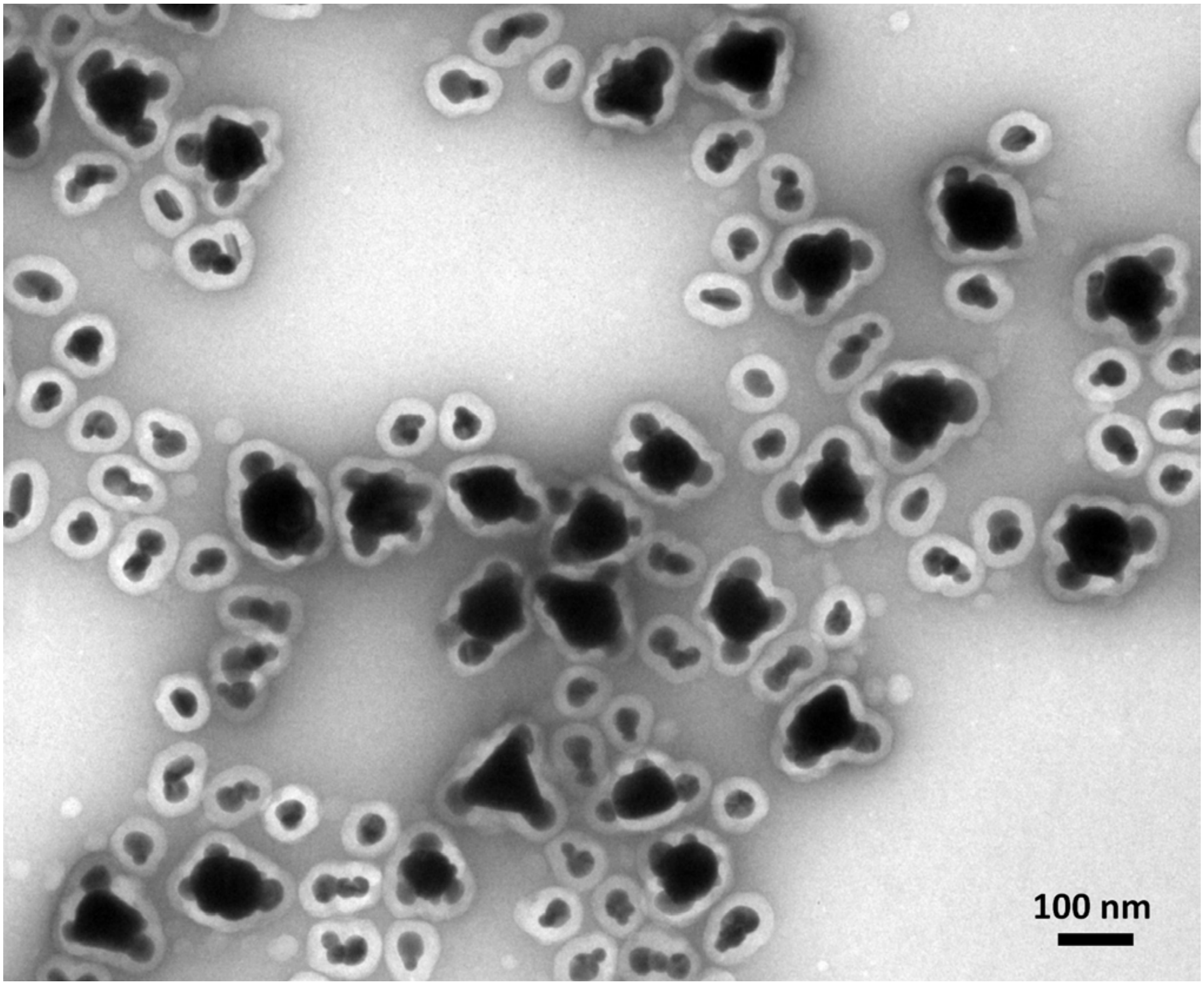

Fig. S8-2 Large-view TEM image of Fig. 5c. A control experiment was carried out with pre-mixed 15, 25, and 70 nm seeds, so that the growth condition is the same for all seeds. The fact that single-island growth (for the 15 nm seeds) and multi-island growth (for the 25 and 70 nm seeds) coexist suggest the same underlying growth principles. The same C_{bulk} should lead to depletion spheres of similar sizes, which would then lead to roughly equal spacing for all seeds. Since the spacing is too large for the 15 nm seeds, the formation of one island prohibits any subsequent nucleation site.

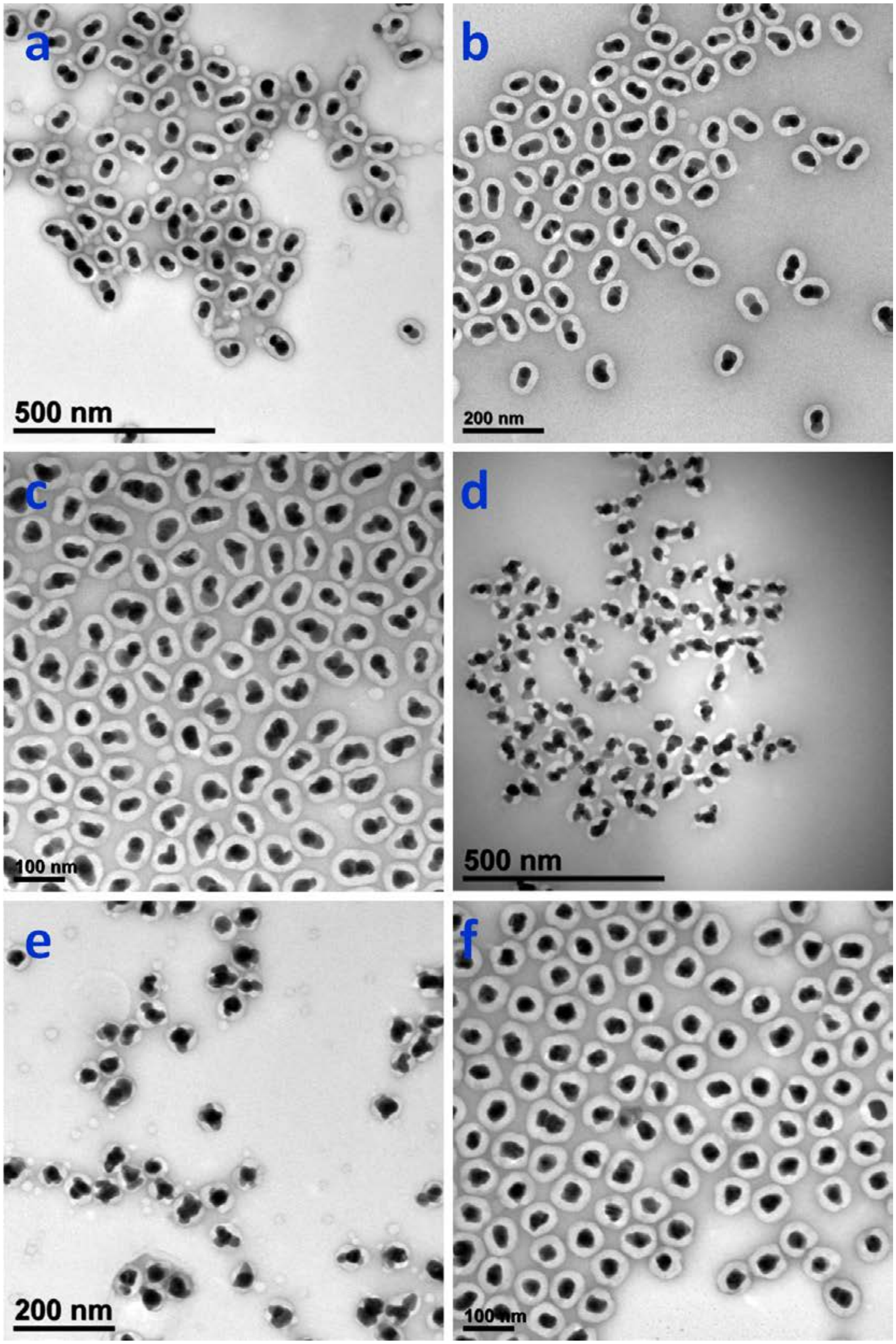

Fig. S9 Growth of Ag islands on 25 nm Au seed nanoparticles under different NaOH concentration: (a) 0.11 mM; (b) 0.15 mM; (c) 0.23 mM; (d) 0.27 mM; (e) 0.34 mM and (f) 0.38 mM. All other conditions were kept the same: [MBIA] in pre-incubation is 20 µM, $[HQ]_{final}$ = 0.74 mM; $[AgNO_3]_{final}$ = 0.37 mM.

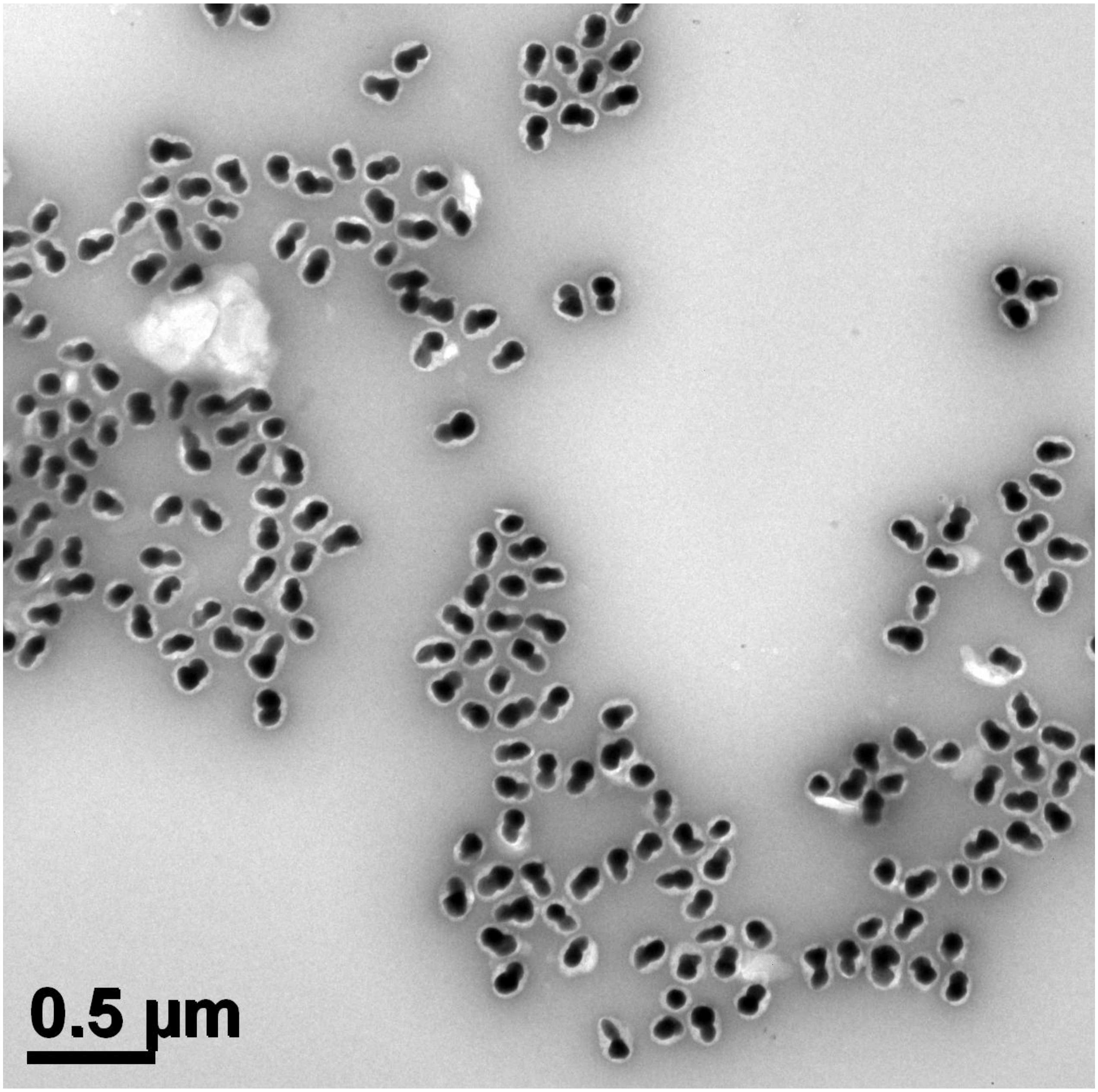

Fig. S10 Large-view TEM image of the Au-Ag Janus nanoparticles, the same sample as shown in Fig. 2a.

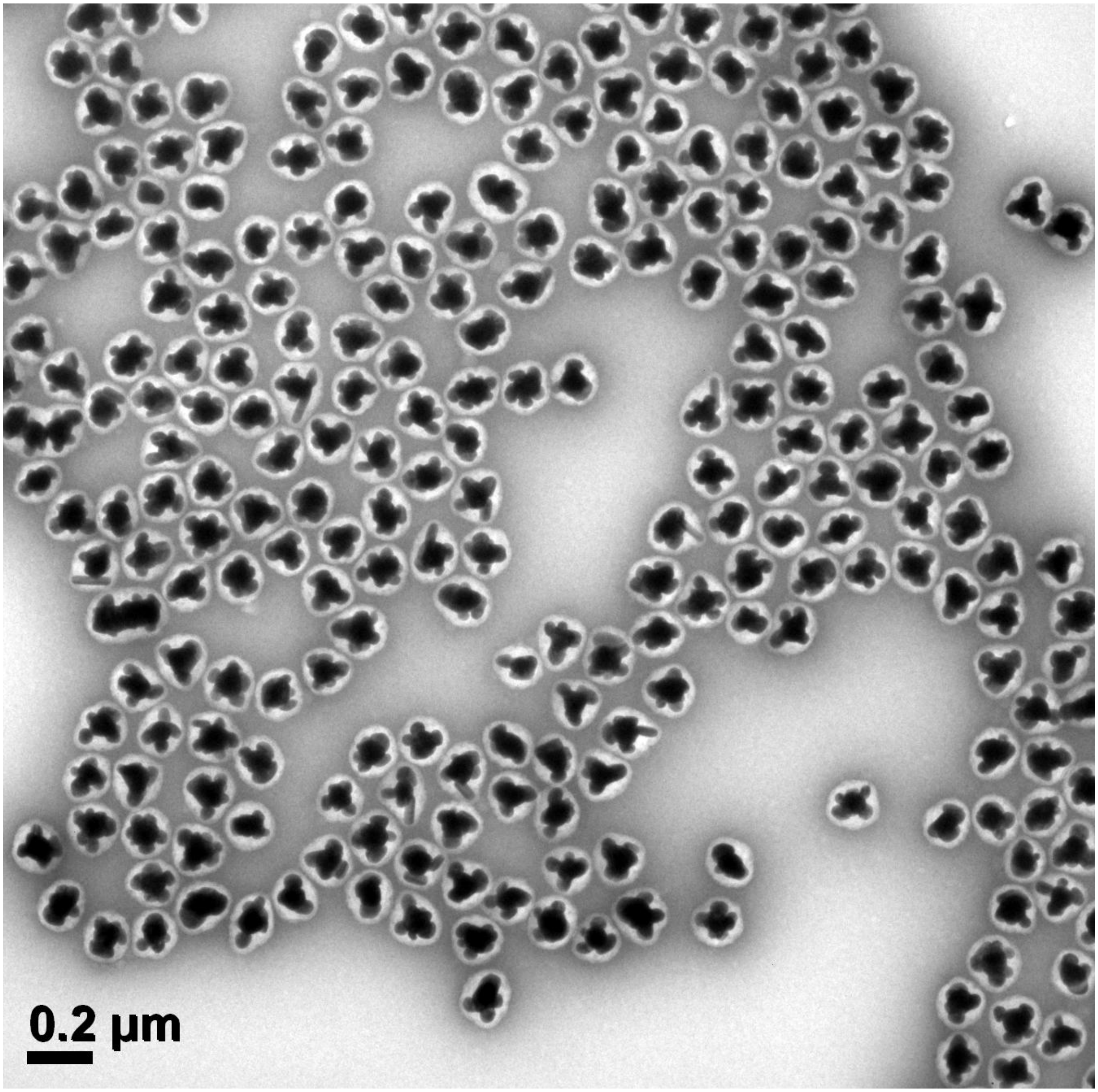

Fig. S11 Large-view TEM image of the Au-Ag satellite nanoparticles, the same sample as shown in Fig. 2c.

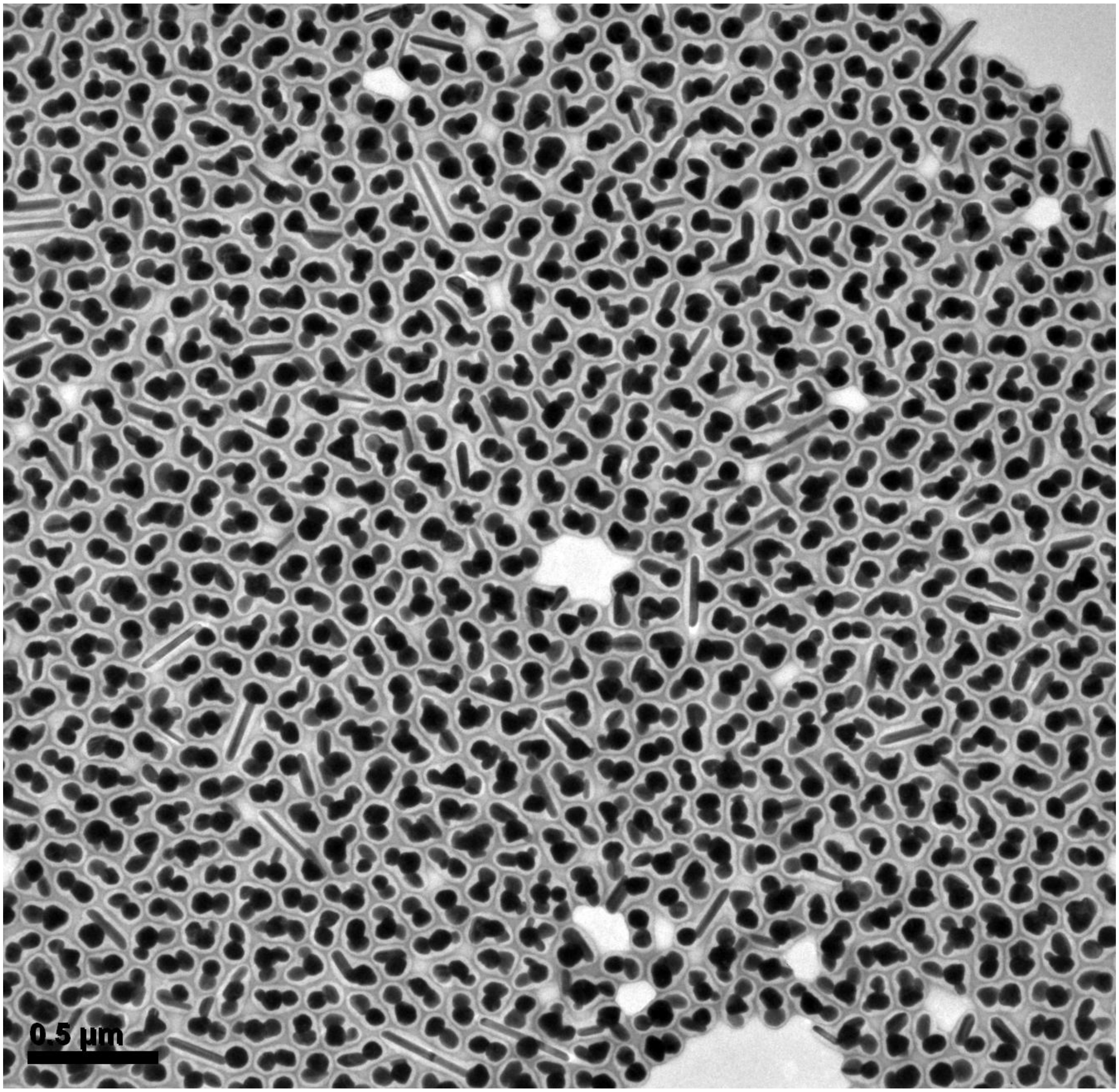

Fig. S12 Large-view TEM image of the Au-Ag hybrid nanoparticles, the same sample as shown in Fig. 3a.

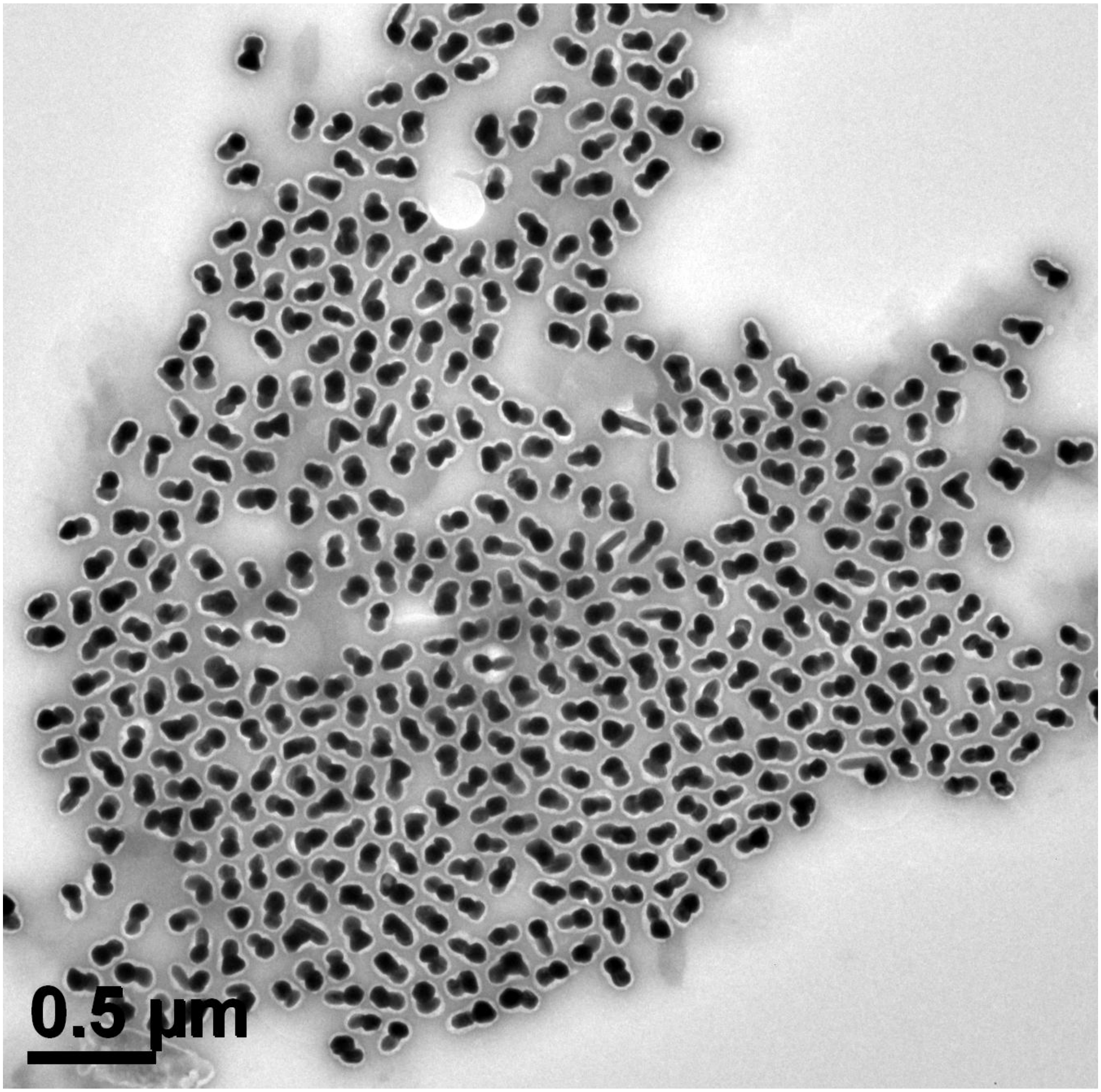

Fig. S13 Large-view TEM image of the Au-Ag Janus nanoparticles (the same sample as shown in Fig. 3a) synthesized under $[HQ]_{final}$ 1.6 mM.

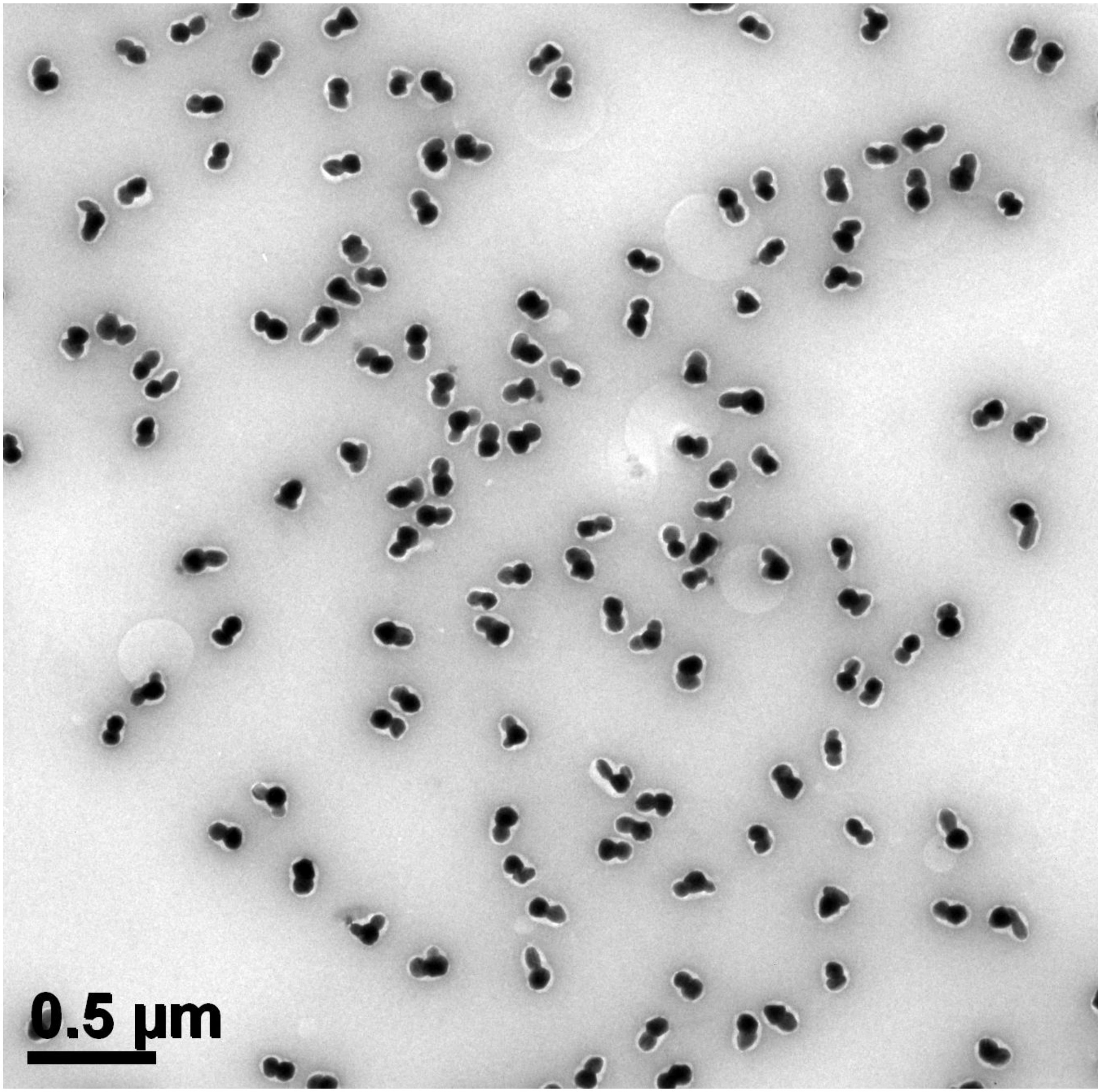

Fig. S14 Large-view TEM image of the Au-Ag Janus nanoparticles (not shown in the main text) synthesized under $[HQ]_{final}$ 3.3 mM.

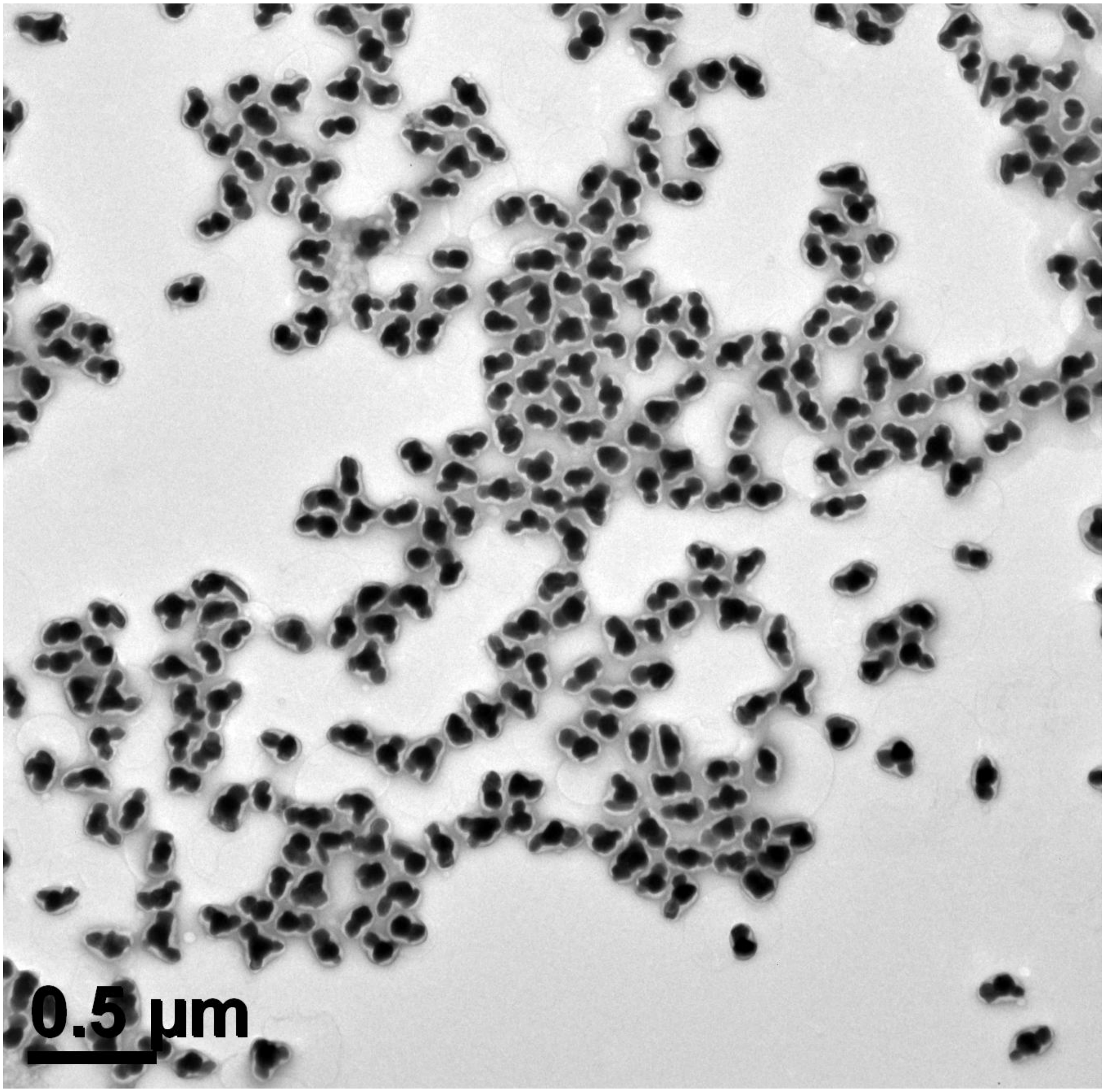

Fig. S15 Large-view TEM image of the Au-Ag hybrid nanoparticles (the same sample as shown in Fig. 3c) synthesized under $[HQ]_{final}$ 6.6 mM.

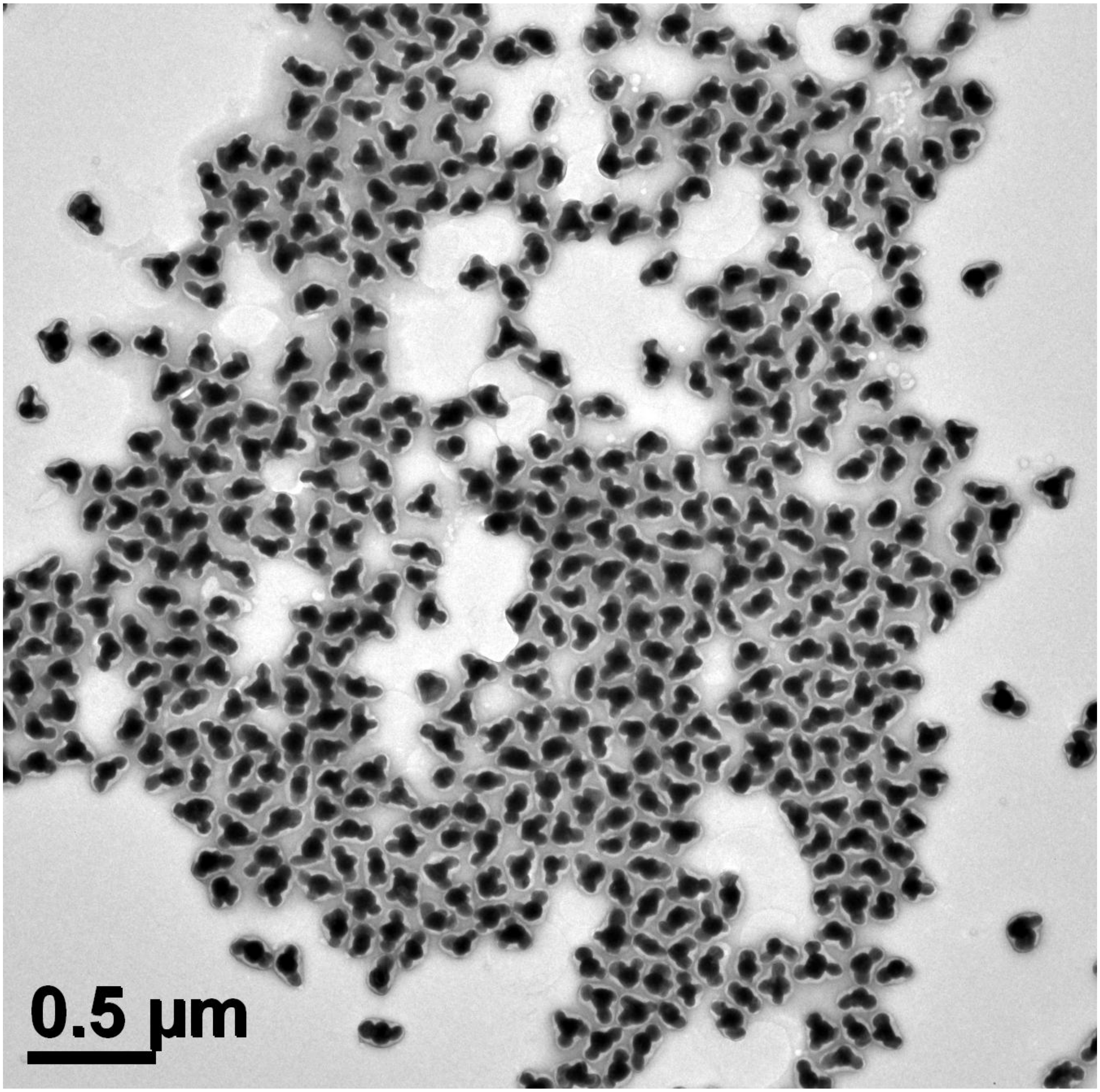

Fig. S16 Large-view TEM image of the Au-Ag satellite nanoparticles (not shown in the main text) synthesized under $[HQ]_{final}$ 9.8 mM.

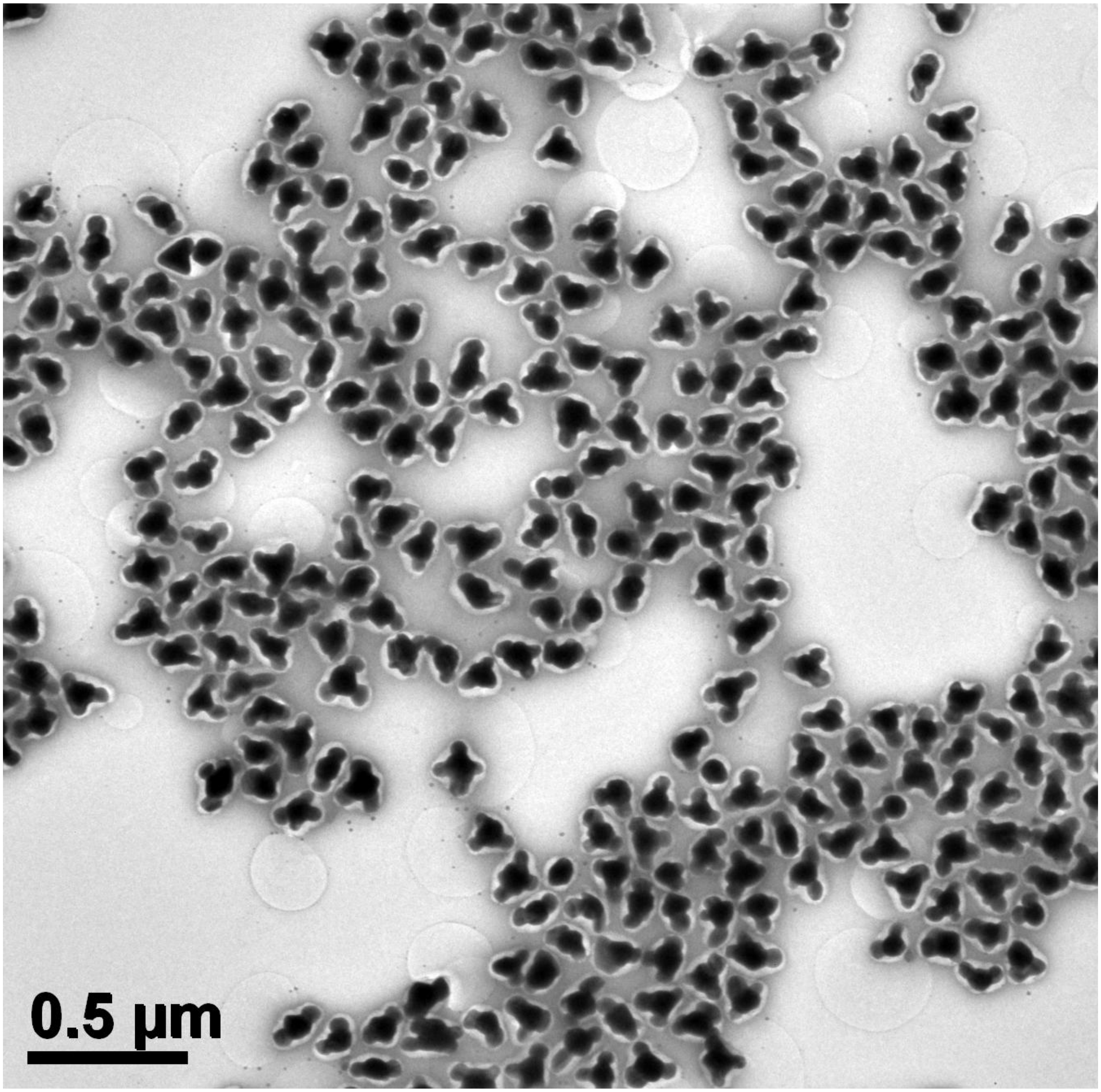

Fig. S17 Large-view TEM image of the Au-Ag satellite nanoparticles (the same sample as shown in Fig. 3e) synthesized under $[HQ]_{final}$ 13.2 mM.

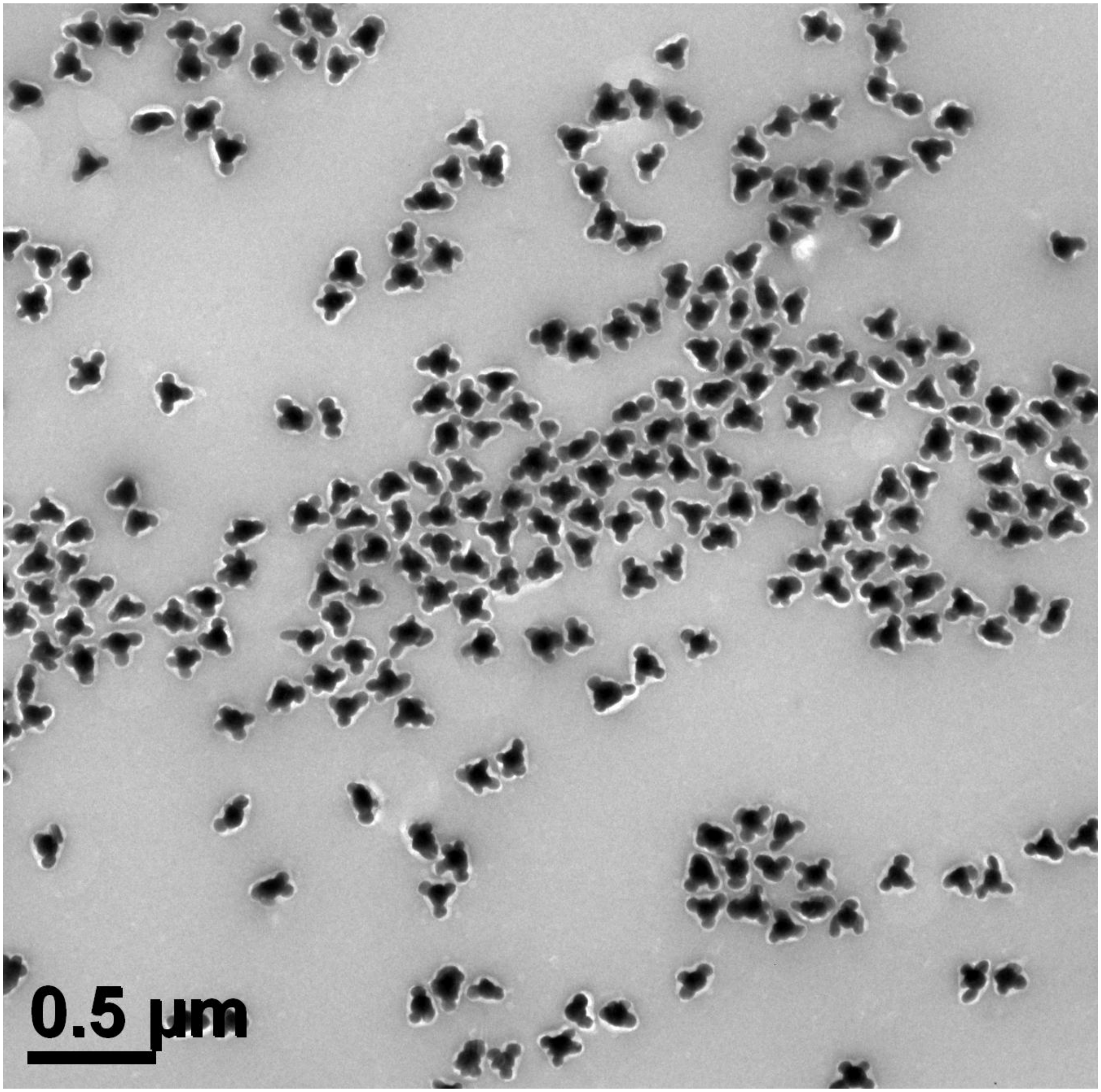

Fig. S18 Large-view TEM image of the Au-Ag satellite nanoparticles (the same sample as shown in Fig. 3g) synthesized under $[HQ]_{final}$ 19.9 mM.

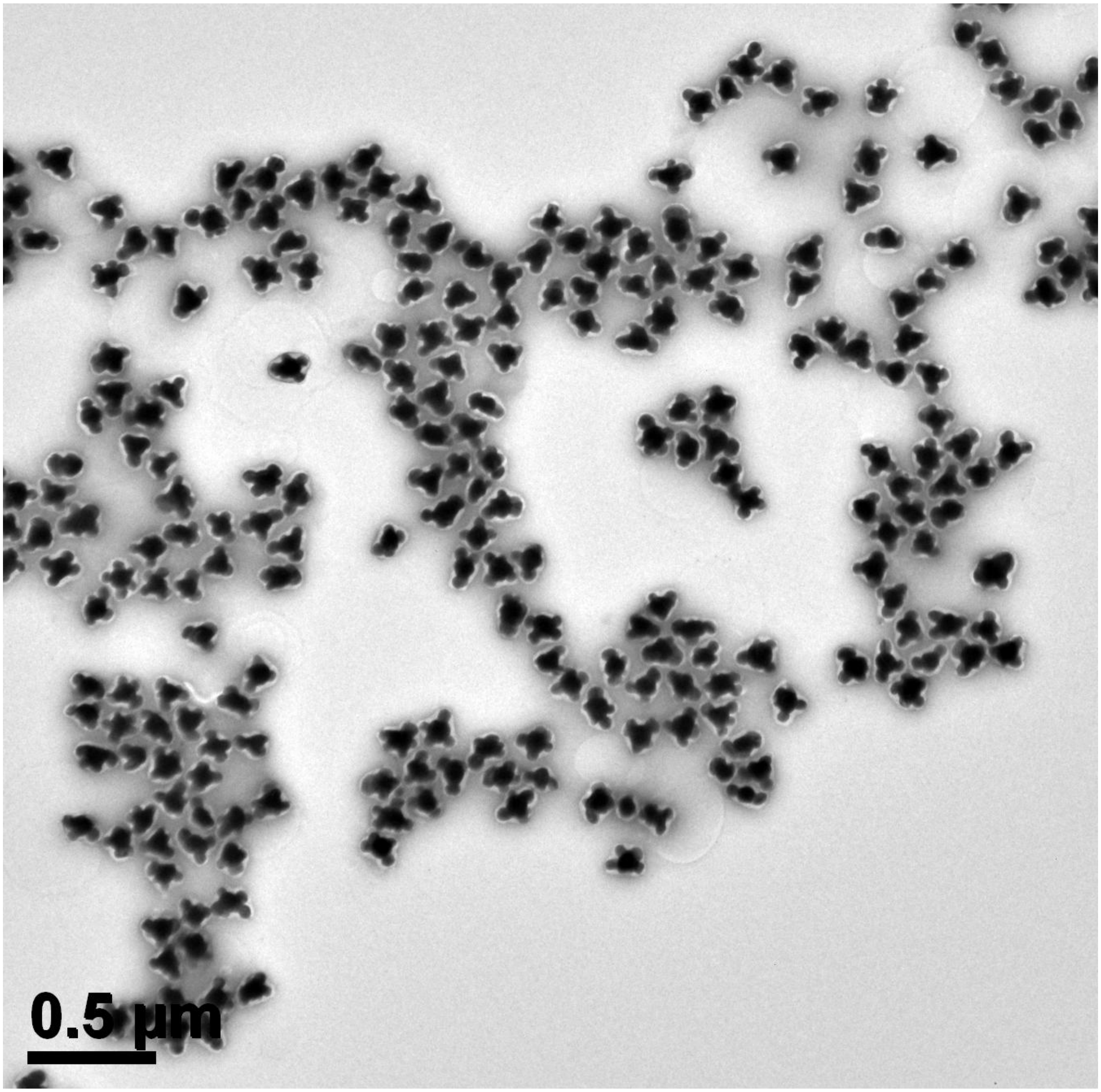

Fig. S19 Large-view TEM image of the Au-Ag satellite nanoparticles (not shown in the main text) synthesized under $[HQ]_{final}$ 26.3 mM.

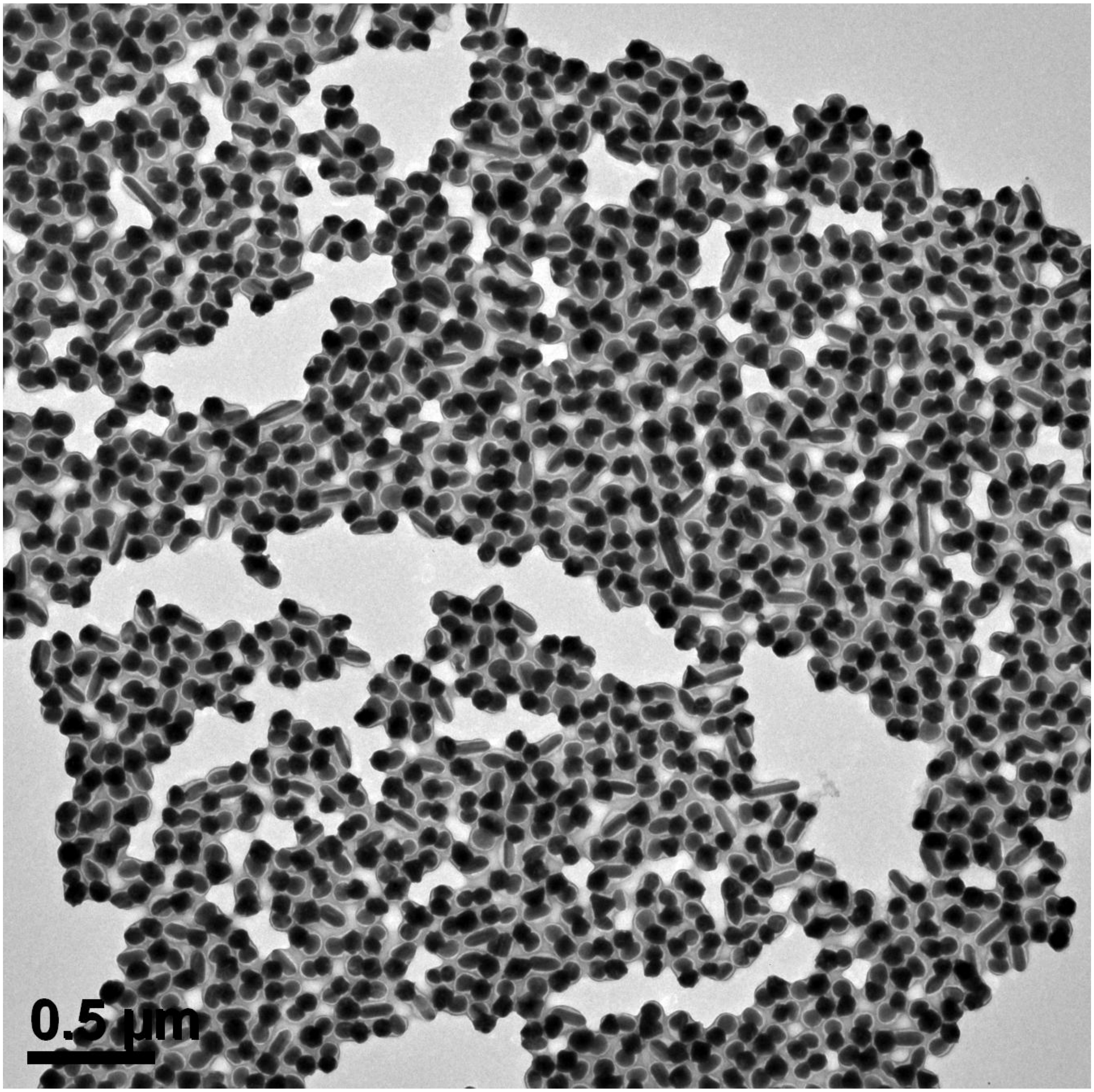

Fig. S20 Large-view TEM image of the Au-Ag Janus nanoparticles (not shown in the main text) synthesized under $[NaOH]_{final}$ 0.09 mM.

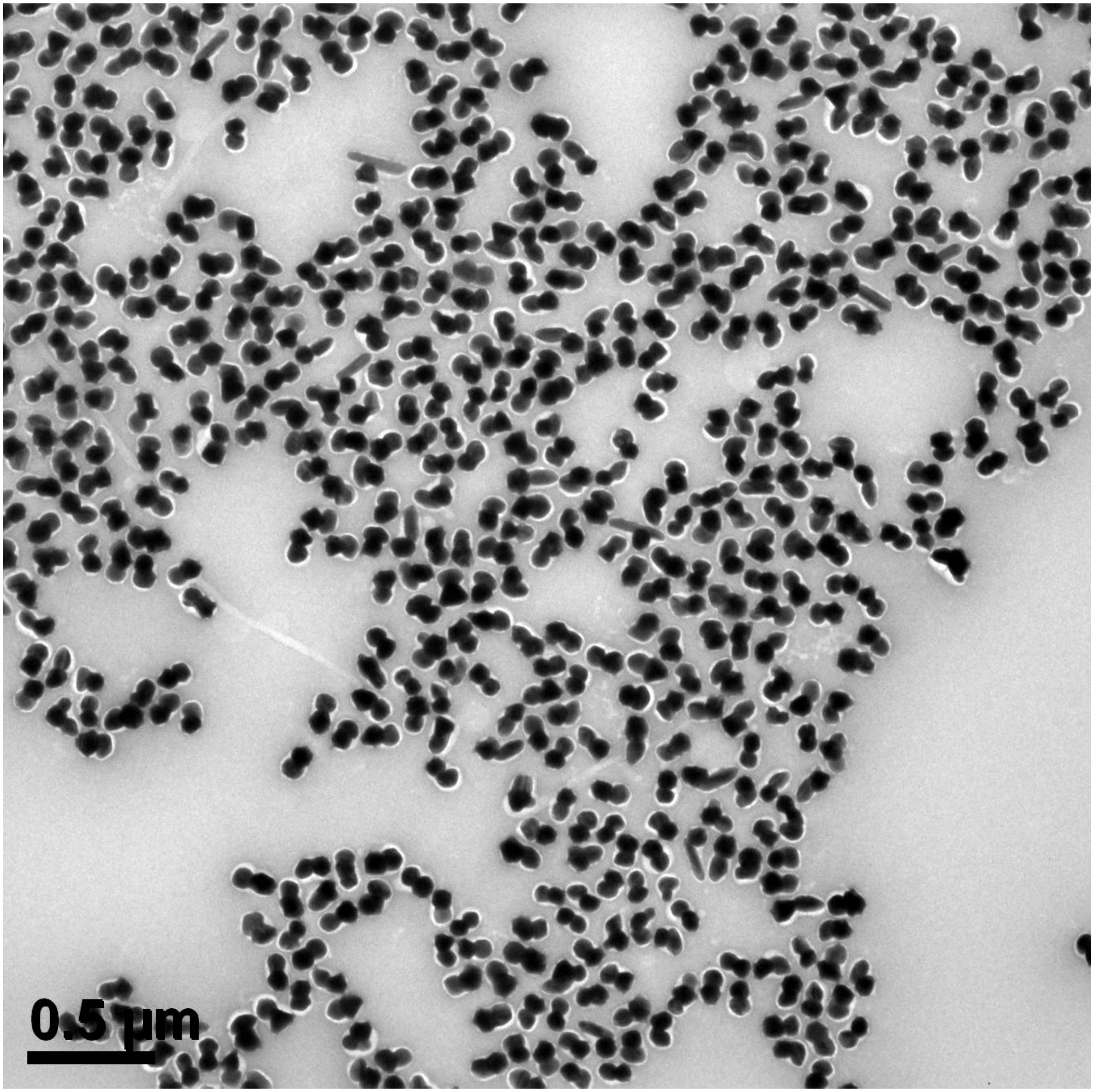

Fig. S21 Large-view TEM image of the Au-Ag Janus nanoparticles (the same sample as shown in Fig. 3i) synthesized under $[NaOH]_{final}$ 0.18 mM.

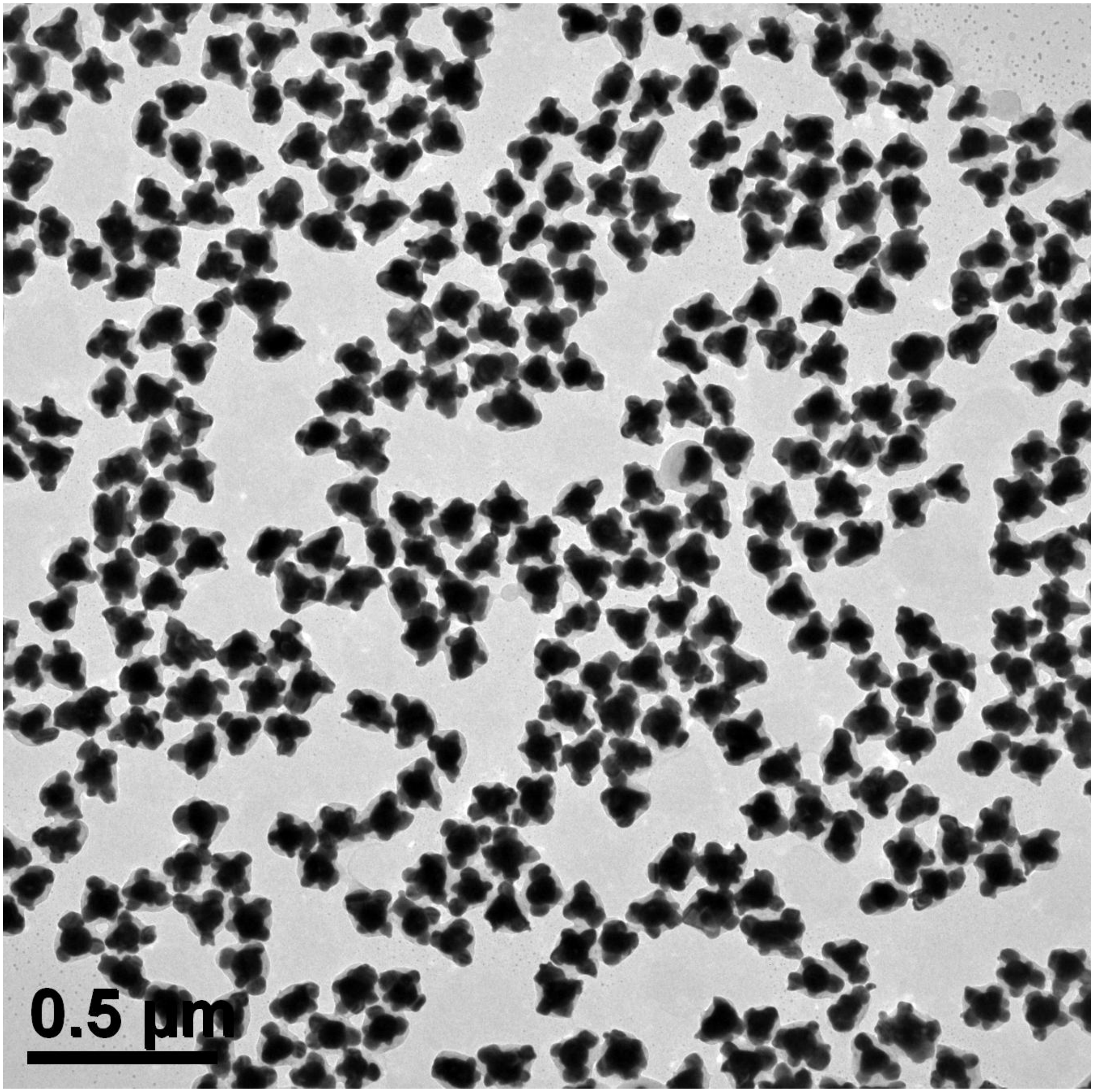

Fig. S22 Large-view TEM image of the Au-Ag satellite nanoparticles (not shown in the main text) synthesized under $[NaOH]_{final}$ 0.27 mM.

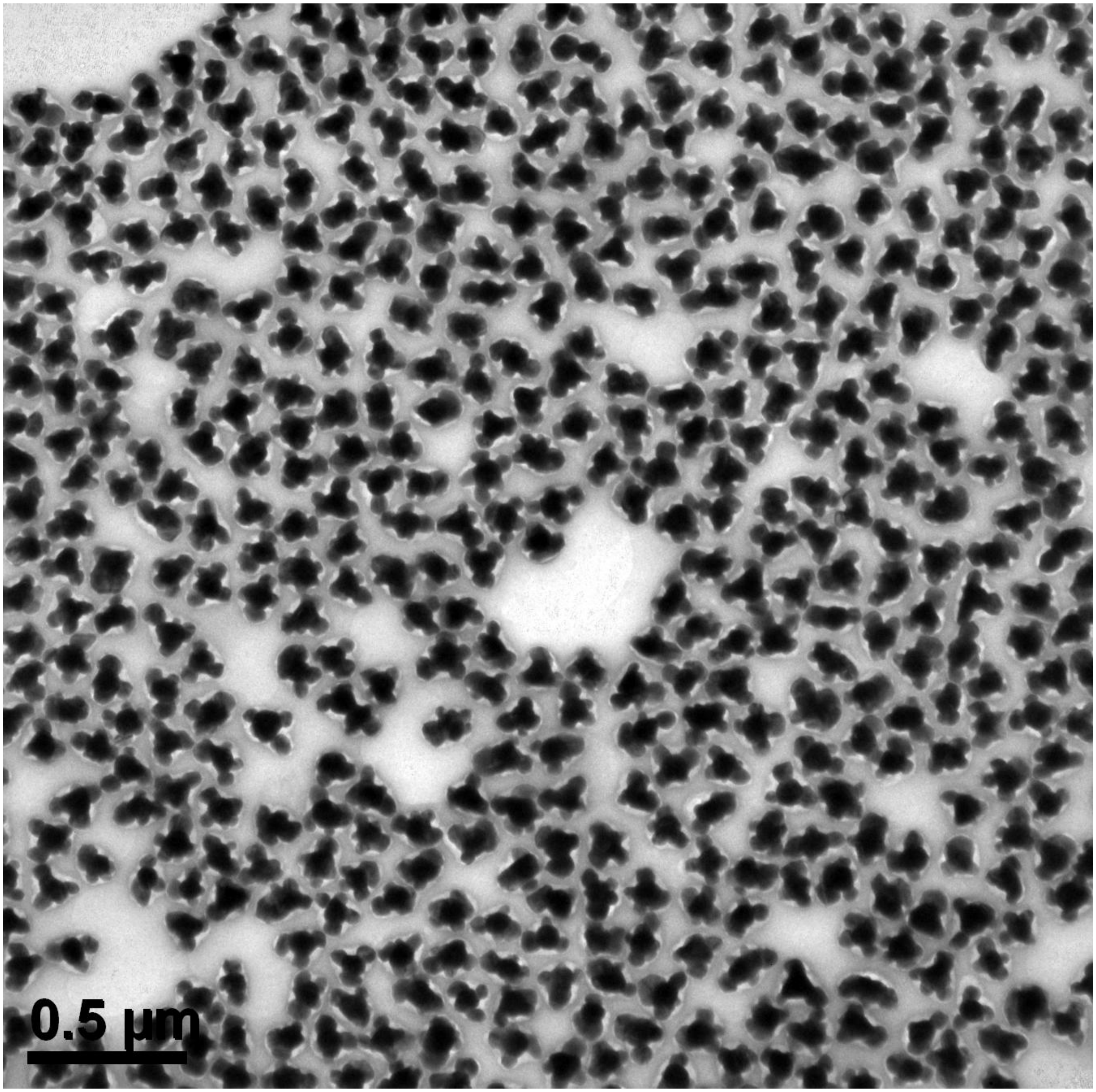

Fig. S23 Large-view TEM image of the Au-Ag satellite nanoparticles (the same sample as shown in Fig. 3j) synthesized under $[NaOH]_{final}$ 0.36 mM.

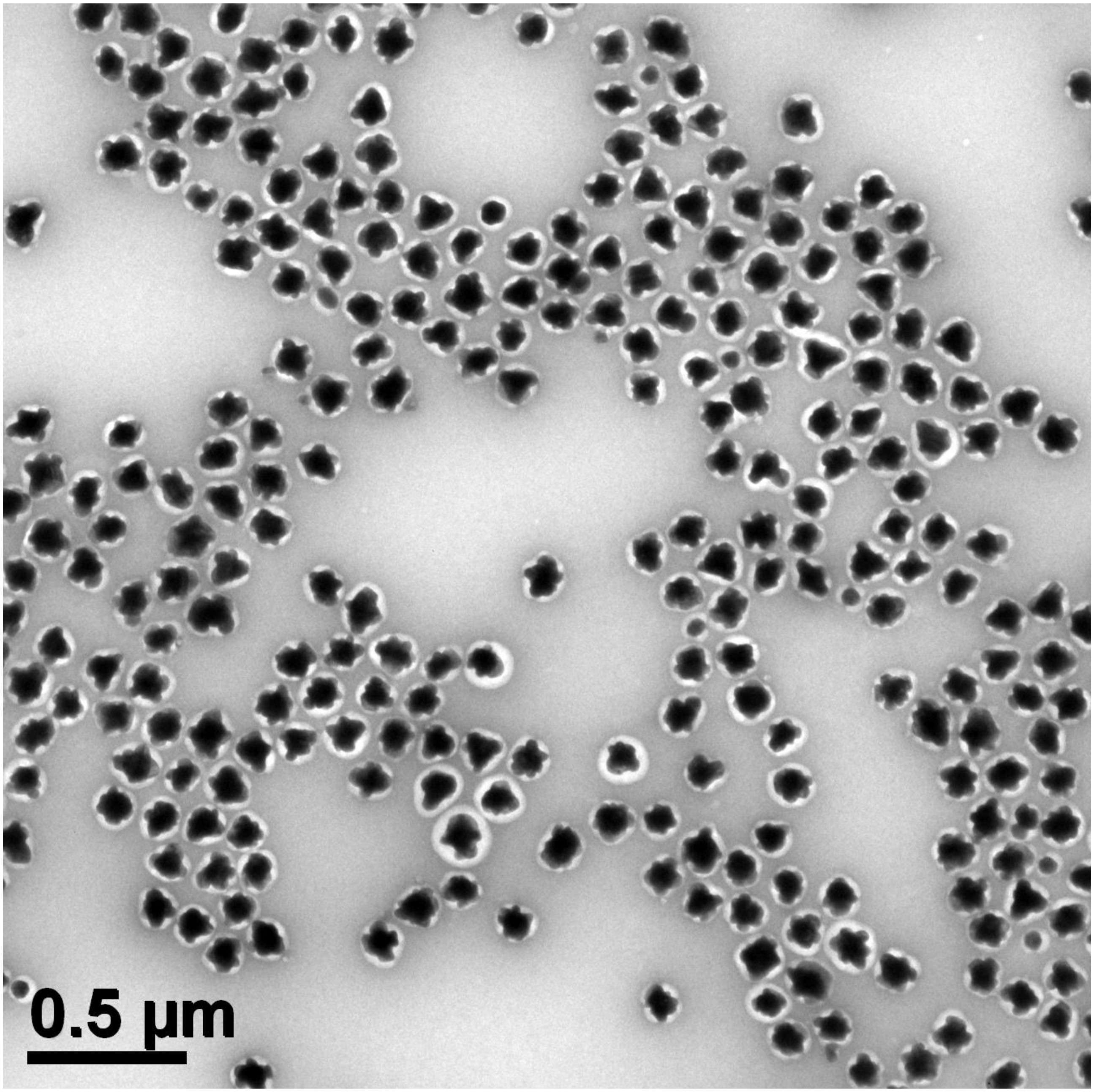

Fig. S24 Large-view TEM image of the Au-Ag satellite nanoparticles (the same sample as shown in Fig. 3k) synthesized under $[NaOH]_{final}$ 0.54 mM.

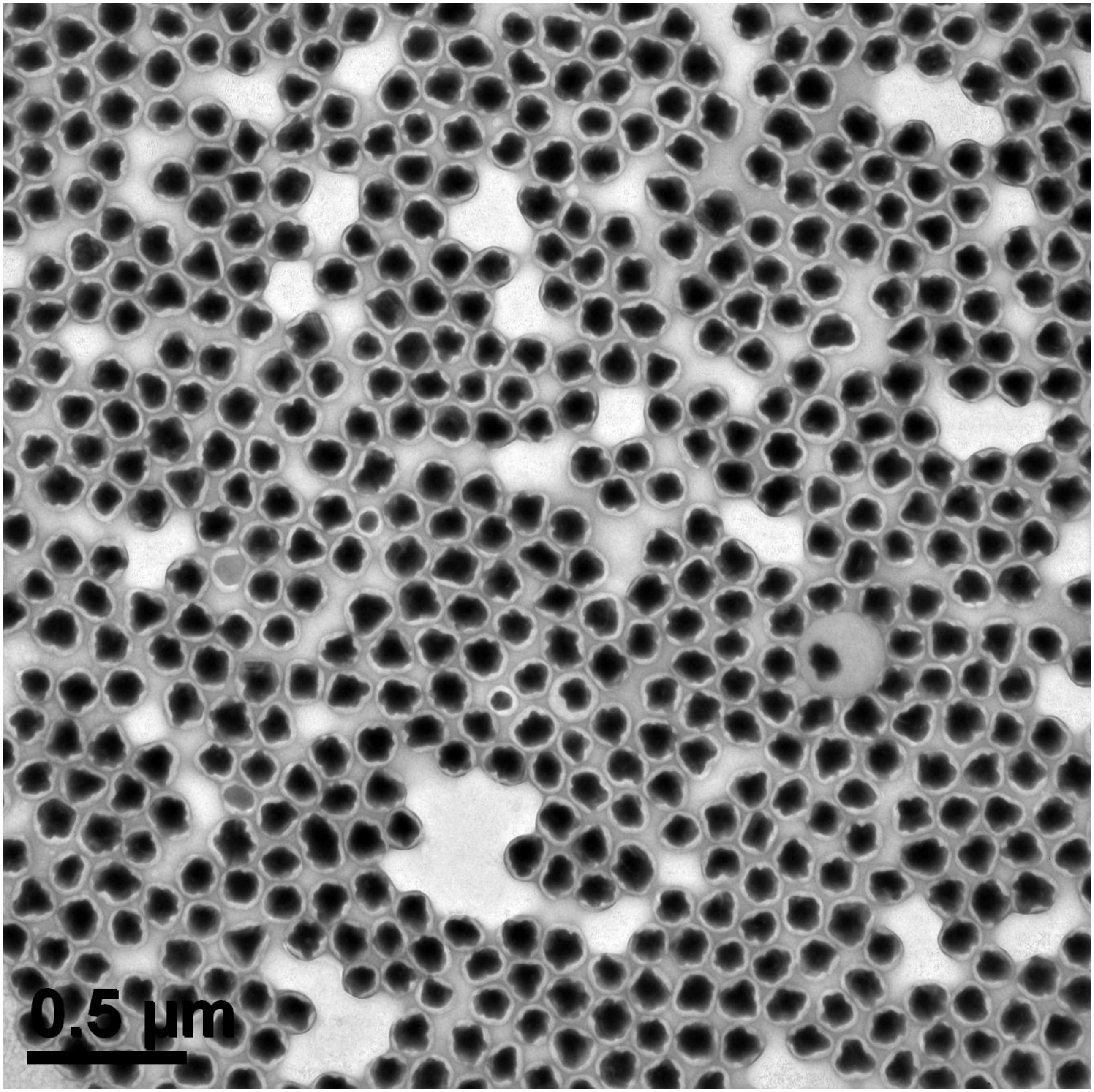

Fig. S25 Large-view TEM image of the Au-Ag satellite nanoparticles (similar to core-shell structure with rough shell surface), the same sample as shown in Fig. 3l synthesized under $[NaOH]_{final}$ 0.72 mM.

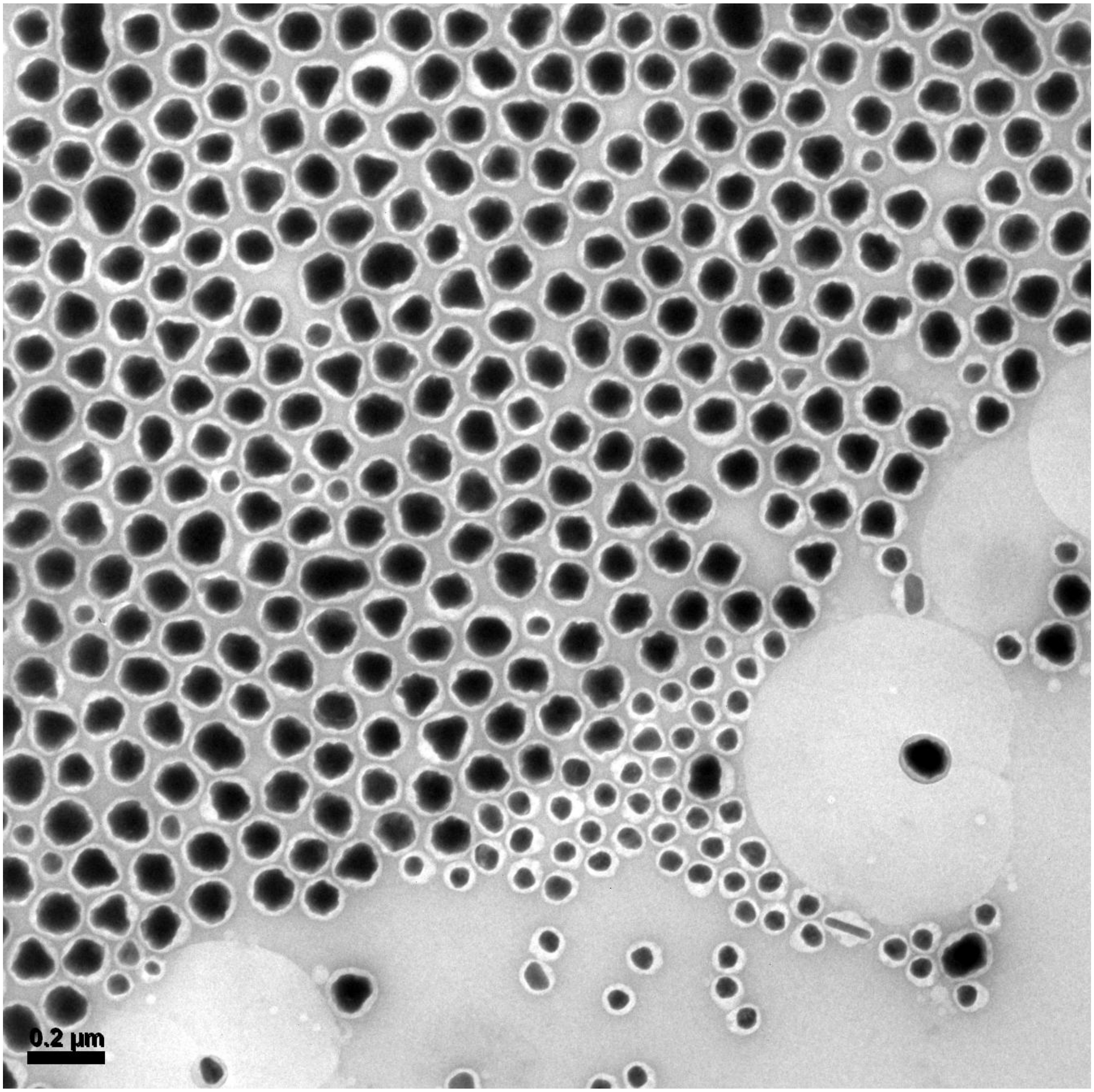

Fig. S26 Large-view TEM image of the Au-Ag satellite nanoparticles (similar to core-shell structure with rough shell surface, not shown in the main text) synthesized under $[NaOH]_{final}$ 0.86 mM. Small pure Ag nanoparticles were observed, which should form due to the fast reduction induced homogeneous nucleation and growth of free Ag nanoparticles.

References:

1. G. Frens, *Nature*, 1973, **241**, 20-22.
2. Y. Feng, J. He, H. Wang, Y. Y. Tay, H. Sun, L. Zhu and H. Chen, *J. Am. Chem. Soc.*, 2012, **134**, 2004-2007.
